# Perturbation of Chromatin Structure Globally Affects Localization and Recruitment of Splicing Factors

**DOI:** 10.1371/journal.pone.0048084

**Published:** 2012-11-12

**Authors:** Ignacio E. Schor, David Llères, Guillermo J. Risso, Andrea Pawellek, Jernej Ule, Angus I. Lamond, Alberto R. Kornblihtt

**Affiliations:** 1 Laboratorio de Fisiología y Biología Molecular, Departamento de Fisiología, Biología Molecular y Celular, Facultad de Ciencias Exactas y Naturales, Universidad de Buenos Aires, Instituto de Fisiología, Biología Molecular y Neurociencias, Consejo Nacional de Investigaciones Científicas y Técnicas, Buenos Aires, Argentina; 2 Dundee Centre for Gene Regulation and Expression, University of Dundee, Dundee, Scotland, United Kingdom; 3 Laboratory of Molecular Biology, Medical Research Council, Cambridge, England, United Kingdom; Institute of Genetics and Molecular and Cellular Biology, France

## Abstract

Chromatin structure is an important factor in the functional coupling between transcription and mRNA processing, not only by regulating alternative splicing events, but also by contributing to exon recognition during constitutive splicing. We observed that depolarization of neuroblastoma cell membrane potential, which triggers general histone acetylation and regulates alternative splicing, causes a concentration of SR proteins in nuclear speckles. This prompted us to analyze the effect of chromatin structure on splicing factor distribution and dynamics. Here, we show that induction of histone hyper-acetylation results in the accumulation in speckles of multiple splicing factors in different cell types. In addition, a similar effect is observed after depletion of the heterochromatic protein HP1α, associated with repressive chromatin. We used advanced imaging approaches to analyze in detail both the structural organization of the speckle compartment and nuclear distribution of splicing factors, as well as studying direct interactions between splicing factors and their association with chromatin *in vivo*. The results support a model where perturbation of normal chromatin structure decreases the recruitment efficiency of splicing factors to nascent RNAs, thus causing their accumulation in speckles, which buffer the amount of free molecules in the nucleoplasm. To test this, we analyzed the recruitment of the general splicing factor U2AF65 to nascent RNAs by iCLIP technique, as a way to monitor early spliceosome assembly. We demonstrate that indeed histone hyper-acetylation decreases recruitment of U2AF65 to bulk 3′ splice sites, coincident with the change in its localization. In addition, prior to the maximum accumulation in speckles, ∼20% of genes already show a tendency to decreased binding, while U2AF65 seems to increase its binding to the speckle-located ncRNA MALAT1. All together, the combined imaging and biochemical approaches support a model where chromatin structure is essential for efficient co-transcriptional recruitment of general and regulatory splicing factors to pre-mRNA.

## Introduction

Several molecular events related to the flow of the genetic information occur in the eukaryotic nucleus. These events depend on the interplay between proteins and nucleic acids that do not act in isolation from other molecules, but generally associate in large complexes that harbor many molecular functions. In this way distinct steps in nuclear processes can be coupled with each other, thereby increasing efficiency. A relevant example is the coupling between the transcription of genes to form nascent pre-mRNA and the simultaneous processing of these nascent transcripts into mature mRNA [1], [2], [3], [4].

All steps of pre-mRNA processing, including capping, splicing and cleavage/poly-adenylation, are known to occur co-transcriptionally [5], [6], although to a different degree depending on the gene. In the case of splicing, organisms as divergent as mammals and yeasts show splicing factor recruitment as soon as their binding sites in the pre-mRNA have been transcribed [7], [8], [9]. Such co-transcriptional RNA processing is, however, not strict, especially considering that the sequence heterogeneity of splice sites found in mammals implies a high variability of recruitment efficiencies. The presence of splice sites whose sequences fall apart from the consensus or are partially obstructed by RNA secondary structures can allow alternative splicing events [10], [11]. However, many constitutive exons also have seemingly weak splice sites, implying that other factors are needed for their efficient definition. These extra factors that participate in both constitutive and alternative splicing are typically auxiliary proteins (*trans* acting factors), such as serine-arginine rich (SR) proteins that bind to sequences in the pre-mRNA (*cis* elements known as splicing enhancers or splicing silencers) [12], [13], [14].

Nuclear architecture is another important factor contributing to the efficiency and specificity of nuclear functions. The nucleus is not a homogenous compartment where molecules diffuse freely, but is rather organized into distinct nuclear compartments [15], [16]. This compartmentalization helps to separate molecular functions within the nucleus, even when these different functions may share molecular actors. It also contributes to reactions efficiency by increasing the local concentration of important components. One example of sub-nuclear compartments in mammalian cells is the interchromatin granule compartment, usually called nuclear speckles or interchromatin granule clusters [17]. These nuclear bodies, normally revealed by immunostaining against the SR protein SRSF2 (previously known as SC35), localize in chromatin-free regions and are enriched in several splicing factors involved in constitutive and alternative splicing. The fact that inhibiting either transcription [18], [19], or pre-mRNA splicing [20] leads to an accumulation of splicing factors in speckles strongly argues that this compartment works as a storage/recycling station rather than as a place where splicing and transcription actually take place. However, active genes are often found at the periphery of speckles [21], [22]. This suggests that accumulation in these granules can assist recruitment more efficiently than relying on passive diffusion throughout the entire nucleoplasm. Consistent with this notion, splicing factors move from speckles to transcription sites upon gene activation [19]. How splicing factors are efficiently recruited to the splice sites to assemble the spliceosome is still a matter of debate, as is what governs their partitioning between speckle-localized, free and splicing-committed proteins. A common feature of the nuclear dynamic assembly of macromolecular complexes seems to be seeding by RNA molecules, such as the nascent pre-mRNA in the case of the spliceosome and nuclear-retained noncoding RNAs such as NEAT1 for paraspeckles [23], [24]. Speckles contain the ncRNA MALAT1, which modulates the localization of several splicing factors, although it seems not to be necessary for the speckle structure to form [25], [26].

Chromatin is now thought to be another important player in the efficient co-transcriptional recognition of splice sites [27], [28], [29], [30]. Different lines of evidence support an important role of chromatin structure in the coupling between transcription and splicing in mammalian cells. First, experiments using viral systems, reporter plasmid minigenes and endogenous genes have shown that compaction of chromatin is correlated with more frequent inclusion of alternative splicing events [31], [32], [33], [34], [35]. Second, some histone tail modifications can assist the recruitment of general and regulatory splicing factors to nascent transcripts through adaptor proteins, hence increasing spliceosome assembly and/or regulating alternative splicing patterns [36], [37]. Third, several reports have determined that nucleosomes are preferentially positioned over exons with respect to introns [38], [39], [40], [41], [42], and this positioning seems to be higher for exons with weak splice sites and exons flanked by large introns [39], [42], suggesting a selective pressure which may act to ensure recognition of difficult exons in the large intron environment of many mammalian genes. Despite this accumulated evidence on the influence of chromatin on splicing, no study assessing the relevance of chromatin structure on the general function of splicing factors has been conducted so far.

Here, we report that relaxation of chromatin structure, promoted by different treatments in several mammalian cell lines, consistently results in depletion of pre-mRNA splicing factors from nucleoplasm and accumulation in nuclear speckles. We demonstrate that this nuclear redistribution is due to general impairment of splicing function and not caused by global transcription inhibition or post-translational modification of the splicing factors. Based on the fact that chromatin relaxation does not affect dynamics of splicing factors, we propose a model describing the relationship between the subnuclear organization of splicing factors and chromatin structure. According to this model, the perturbation of chromatin structure impairs the recruitment of splicing factors to nascent RNAs. We tested this hypothesis by assessing early spliceosome recruitment through U2AF65 iCLIP analysis, which confirms the reduced recruitment and shows that splicing factors are redirected to low affinity binding sites present in abundant ncRNAs such as speckles-located MALAT1 and paraspeckles-located NEAT1.

## Results and Discussion

### Relaxation of Chromatin Structure causes Accumulation of Splicing Factors in Nuclear Speckles

**Figure 1:**
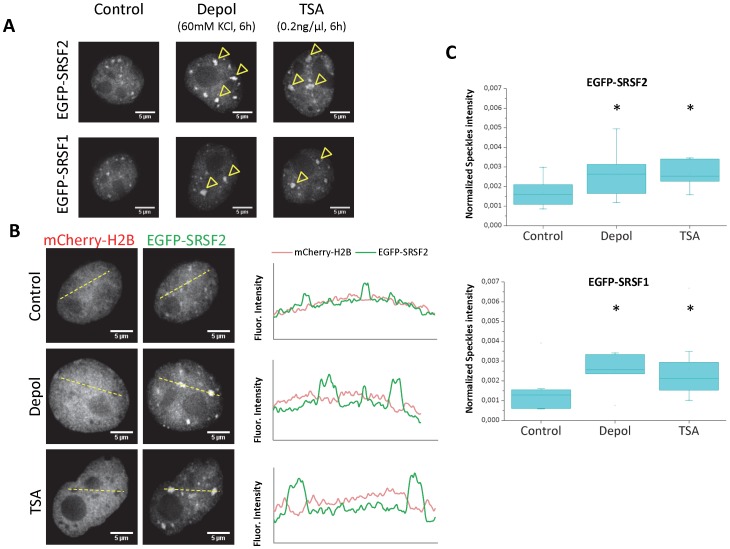
Membrane potential depolarization and TSA treatment of neuroblastoma cells cause splicing factors accumulation in speckles. (A) N2a cells were transiently transfected with plasmids encoding SRSF2 or SRSF1 splicing factors fused to EGFP, along with a plasmid encoding histone H3 fused to mCherry. After one day, cells were either treated for 6 hours with 0.2 ng/µl TSA (TSA), 60 mM KCl (DEPOL) or left untreated (CONTROL). Enlarged nuclear speckles in N2a cells containing the splicing factors in DEPOL or TSA-treated cells are marked by yellow arrowheads. Scale bars, 5 µm. (B) Analysis of intensity profile of EGFP-SRSF2 and mCherry-H2B signals across a line (yellow dotted lines) for a representative cell for each experimental condition. SRSF2 profile shows higher and wider peaks in DEPOL and TSA cells, corresponding to enlargement of nuclear speckles. No intensity changes are observed for mCherry-H2B profiles. The drop in mCherry-H2B signal at the SRSF2 peaks is typical of inter-chromatin granules. Scale bars, 5 µm. (C) Statistical analysis of splicing factor enrichment in speckles. Signal of EGFP-SRSF2 (top) and EGFP-SRSF1 (bottom) in speckles increases both in response to depolarization and TSA treatments. Intensity of splicing factor in all speckles of a focal plane was calculated for individual cells using automatic threshold and particle analysis (see Materials and Methods). The total integrated density of speckles particles was normalized by total integrated density of the cell. For EGFP-SRSF2, 10 cells (Control), 8 cells (Depol) and 11 cells (TSA) were analyzed. For EGFP-SRSF1, 10 cells (Control), 7 cells (Depol) and 10 cells (TSA) were analyzed. * means significant differences between treated and control cells, using Mann-Whitney U test (p = 0.023 in Depol and 0.0044 in TSA for EGFP-SRSF2; p = 0.036 in Depol and 0.031 in TSA for EGFP-SRSF1).

In a previous report [35] we showed that treatment of N2a murine neuroblastoma cells with high extracellular potassium concentration promotes histone H3 lysine 9 acetylation in an intragenic region of the NCAM gene, consequently affecting alternative splicing of exon 18. This effect can be mimicked by treatment with the histone deacetylases inhibitor trichostatin A (TSA). As depolarization treatment also causes a general increase in histone H3 and H4 acetylation [35], we hypothesized that its effect on splicing could be more general than the modulation of this particular event. Since we wanted to analyze splicing mechanisms not biased to any particular gene or exon, we monitored the distribution of two SR proteins that participate in alternative and constitutive splicing: SRSF2 and SRSF1. N2a cells transiently expressing the fluorescent fusion proteins EGFP-SRSF2 or EGFP-SRSF1 and treated for 6 hours with either high KCl concentrations (DEPOL) or TSA, showed an increase in the accumulation of the tagged splicing factors in enlarged speckles in comparison to control cells (Fig. 1A, arrowheads). To quantify this accumulation, we measured the fluorescence intensity across a line in representative cell nuclei. The resulting profiles showed that, after depolarization or TSA treatment, EGFP-SRSF2 signal had higher and wider peaks corresponding to the enlarged speckles (Fig. 1B). Similar profiles were observed for EGFP-SRSF1 (not shown). As a control, cells were co-transfected with a plasmid encoding mCherry-H2B histone, which showed no change in the overall chromatin localization (Fig. 1B). The only apparent change is a more pronounced H2B valley coincident with SR protein peaks, supporting the conclusion that the splicing factors are accumulated in interchromatin granules. To have a more objective evidence of speckle enlargement upon these treatments, we quantified the speckles-associated signal of EGFP-SRSF2 and EGFP-SRSF1 in several individual cells (Fig. 1C). Depolarization and TSA treatment induced a similar significant increase in the signal of both splicing factors in speckles compared to control N2a cells. Altogether, we showed that an extracellular signal known to trigger histone acetylation and a drug that causes increased acetylation by inhibiting deacetylases activity have a similar effect on the nuclear distribution of these SR proteins, by promoting a conspicuous and quantitative concentration in nuclear speckles.

**Figure 2:**
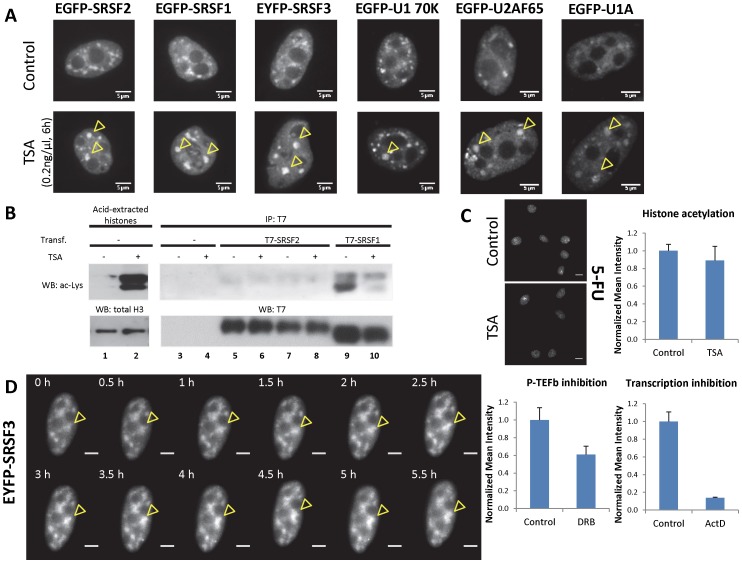
Histone acetylation affects distribution of several splicing factors involved in constitutive and alternative splicing. (A) Several splicing factors tested show accumulation in nuclear speckles after a 6-hour TSA treatment of HeLa cells, including many SR proteins (SRSF2, SRSF1, SRSF3) and proteins of the U1 and U2 complexes (U1 70K, U1A, U2AF65). Top row shows control cells and bottom row shows cells treated with 0.2 ng/µl TSA. Some enlarged nuclear granules containing splicing factors in TSA-treated cells are marked by yellow arrowheads. Scale bars, 5 µm. (B) TSA treatment affects histone but not splicing factor lysine acetylation. HEK-293T cells were transfected with plasmids coding for the indicated tagged proteins (lanes 5–10) or not transfected (lanes 1–4). After one day, cells were incubated for 6 hours with or without 1 µM TSA, lysed and either histones were extracted (lanes 1–2) or proteins immunoprecipitated with antibodies against T7 tag (lanes 3–10). Purified proteins were analyzed by Western blot using an antibody that recognizes acetylated lysines (αAcLys, upper panels). As loading controls, proteins were also detected with antibodies against histone H3 or T7 tag (lower panels). In lanes 1 and 2, the two bands for the αAcLys correspond to histones H3 and H4, showing a dramatic increase of acetylation in both histones upon TSA treatment. In lanes 7 and 8, cells transfected with T7-SRSF2 were treated with the proteasome inhibitor MG132 (see text). (C) Total transcription is not affected after 6 h-TSA treatment. Global levels of nascent RNA in untreated and TSA-treated HeLa cells were measured by *in vivo* 5-FU incorporation and immunostaining with an antibody specific for 5-FU nucleotide. A representative field for each condition is shown. Brighter spots correspond to ribosomal RNA in nucleoli. Scale bars, 10 µm. Quantification of the 5-FU signal in individual cells (see Materials and Methods) shows no significant change in transcription in TSA-treated vs. Control cells (*n* = 15 and 10 respectively). In contrast, treatment with the P-TEFb inhibitor DRB (*n* = 10 for each condition) and with the general transcription inhibitor actinomycin D (*n* = 5 for Control and 6 for ActD) causes a clear decrease in 5-FU incorporation, as expected from transcriptional inhibition.(D) Time course of EYFP-SRSF3 distribution in HeLa cells treated with 0.2 ng/µl TSA. A representative nucleus is shown. Images were acquired every 30 minutes. Yellow arrowheads point to a single speckle where accumulation of the tagged-SR protein is observed. Scale bars, 5 µm.

To investigate the generality of this phenomenon, we transiently transfected human cells of non-neural origin with different splicing factors fused to fluorescent proteins. We analyzed the nuclear distribution of three different SR proteins (SRSF2, SRSF1 and SRSF3, the latter previously known as SRp20) and three constitutive splicing factors (U1 70K, U1A and U2AF65) in HeLa cells. All six splicing factors accumulated in speckles after treatment with TSA for 6 hours (Fig. 2A, arrowheads, and Fig. S1), suggesting that the treatment affects a general step in the splicing process rather than the behavior of specific proteins.

Since TSA could affect the acetylation status of non-histone proteins, a possible explanation was that splicing factors were hyper-acetylated after the treatment and this post-translational modification could explain the observed stronger localization in speckles. In fact, acetylation of SR proteins was already described for SRSF2 [43]. We monitored whether TSA affected the acetylation status of SRSF2 and SRSF1 by transfecting HEK-293T cells with plasmids encoding T7-tagged proteins, immunoprecipitating them and performing Western blot analysis with an antibody that recognizes acetylated lysines (Fig. 2B). To verify that TSA was effective in increasing acetylation levels and to check if the acetyl-Lys antibody worked properly, we purified histones from cells either untreated or treated with TSA. As expected, an increase in the antibody signal was detected in histone extracts from TSA-treated cells (Fig. 2B, lanes 2 vs. 1). In contrast, only a weak acetyl-Lys signal was detected for T7-SRSF2 and only a slightly stronger signal for T7-SRSF1 (lanes 5 and 9 vs. 3). More importantly, no increase in lysine acetylation was detected after TSA treatment for these SR proteins (Fig. 2B, lane 6 vs. 5 and lane 10 vs. 9). Since acetylation of SRSF2 was reported to result in degradation by the proteasome [43], we tested if an increase in the levels of T7-SRSF2 acetylation was revealed after inhibition of proteasome activity by MG132. No changes in the acetylation levels of T7-SRSF2 were detected after MG132 treatment, either in the absence, or presence, of TSA (Fig. 2B, lanes 7–8 vs. 5–6). From these experiments we concluded that the possibility of a TSA-triggered accumulation of splicing factors in speckles due to enhancement of their acetylation levels is unlikely.

As previously mentioned, inhibition of transcription causes the accumulation of splicing factors in speckles. Although TSA is not expected to inhibit transcription, a partial effect on *de novo* RNA synthesis could in theory cause the observed effect. To test this, we measured general RNA synthesis levels by pulse-labeling nascent RNAs with the modified nucleotide 5-fluoruracil (5-FU) and performing immunofluorescence analysis with an antibody that recognizes 5-FU (Fig. 2C). The labeling was distributed all over the nucleus, with some intense foci corresponding to rRNA synthesis in nucleoli. We quantified the mean fluorescence intensity in nuclei from control and TSA-treated HeLa cells and detected no significant differences in the levels of general RNA synthesis (Fig. 2C, Histone acetylation graph). As a proof that measure of 5-FU incorporation can detect both general and pol II-mediated transcriptional inhibition, we performed the same analysis on HeLa cells treated with drugs that inhibit transcription through different mechanisms. The nucleotide analog 5,6-dichloro-1-beta-D-ribofuranosylbenzimidazole (DRB) is known to partially prevent pol II transcription through inhibition of the elongation factor P-TEFb. In our assay, a 2 h treatment with 25 µg/ml DRB caused a ∼40% decrease in 5-FU incorporation (Fig. 2C, P-TEFb inhibition graph), a magnitude comparable to nuclear run-on measurements in cells treated with P-TEFb inhibitors [44]. As expected, a 2 h treatment with 1 µg/ml actinomycin D (ActD), that affects transcription by all RNA polymerases, led to an almost complete inhibition (near 90%) of 5-FU incorporation (Fig. 2C, Transcription inhibition graph), showing the dynamic range of this assay. Finally, to rule out potential general effect of TSA on pol II transcriptional elongation, we performed immunostaining with an antibody against tri-methylated lysine 36 of histone H3 (H3K36me3), a hallmark of actively transcribed regions, on HeLa cells. Treatment with TSA for 2 or 6 hours didn’t reveal any significant alteration of H3K36me3 pattern (Fig. S1). Also, we didn’t detect consistent transcription inhibition of individual genes (not shown). In conclusion, the 5-FU incorporation assay shows no general inhibition of transcription by the TSA treatment while the H3K36me3 staining shows no effect on a pol II elongation-associated mark. These results rule out the possibility that TSA is exerting its effect through inhibition of transcription.

The lack of any TSA effects on splicing factor acetylation and general transcription levels argues against indirect effects being responsible of the observed accumulation of splicing factors in speckles. Moreover, the fact that accumulation in speckles is already high 6 hours after TSA addition suggests that TSA starts exerting its effect immediately and supports a direct effect, rather than an indirect effect, such as changing the patterns of splicing regulators or interference with cell cycle progression. To confirm this, and to make sure that the reorganization occurs within an individual cell, we performed time-lapse experiments on single cells expressing fluorescent fusion splicing factors. As seen in Fig. 2D for EYFP-SRSF3, a gradual increase of speckle fluorescence intensity (Fig. 2D, arrowhead) with respect to the nucleoplasm is already evident at short times, reaching a maximum at around 4 hours.

### Analysis of Endogenous Splicing Factor Distribution and Speckle Structure After Perturbation of Chromatin Structure

**Figure 3:**
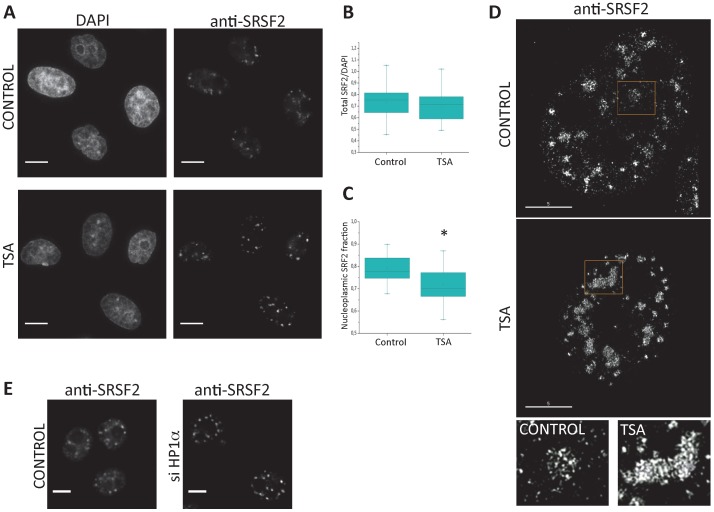
Analysis of endogenous SRSF2 distribution shows that histone acetylation causes splicing factor depletion from nucleoplasm. (A) Immunostaining for endogenous SRSF2 in HeLa cells untreated or treated with TSA (0.2 ng/µl, 6 hours). DNA was stained with DAPI. Visualization of endogenous SRSF2 distribution suggests depletion from nucleoplasm after TSA treatment. Scale bars, 10 µm. (B) Total levels of endogenous SRSF2 do not change upon TSA treatment. Box plot showing similar distribution of SRSF2/DAPI intensities in individual untreated or TSA-treated HeLa cells (26 cells of each type). The SRSF2/DAPI ratio is NOT significantly different between CONTROL and TSA cells (Mann-Whitney U test: p = 0.23; Kolmogov-Smirnov test: p = 0.501). (C) TSA treatment causes depletion of nucleoplasmic SRSF2 fraction. Nucleoplasmic intensity was calculated in the same cells as (B) (see Materials and Methods). Box plot shows nucleoplasmic/total SRSF2 fractions in CONTROL and TSA cells, which further supports the observation of SRSF2 depletion from nucleoplasm after TSA treatment. The nucleoplasmic SRSF2 fraction is significantly reduced in TSA-treated cells compared to control cells (marked by asterisk; Mann-Whitney U test: *p* = 0.0017; Kolmogov-Smirnov test: *p* = 0.018). (D) High resolution imaging analysis of SRSF2 distribution and speckle internal structure in untreated and TSA-treated cells. Control and TSA-treated cells were fixed, immunostained for SRSF2 and analyzed by SIM/OMX microscopy. Upper panels: a representative cell in each condition is shown, where depletion of diffuse SRSF2 in nucleoplasm and concentration in speckles is apparent after TSA treatment. Lower panels: enlarged view of an individual speckle (yellow boxes) from cells in each experimental condition. Scale bars, 5 µm. (E) HP1α knockdown has similar effect than TSA treatment on endogenous SRSF2 distribution. HeLa cells were transfected with control siRNAs or siRNAs against HP1α. After two days, the cells were fixed and stained for endogenous SRSF2. Depletion of nucleoplasmic SRSF2 staining and accumulation in speckles is evidenced, similar to what is seen after TSA treatment (A). Scale bars, 5 µm.

While general transcription is not substantially affected by TSA treatment, it could enhance the overexpression of exogenous fused FPs-splicing factors constructs over time. As a consequence, the excess of splicing factors could cause the impression of concentration in speckles. To verify that overexpression is not responsible for the observed speckle-accumulation of the splicing factors, we analyzed the distribution of endogenous SRSF2 by indirect immunofluorescence in HeLa cells, either untreated, or treated with TSA (Fig. 3A). TSA-induced accumulation of SRSF2 in speckles was also seen for the endogenous protein, although the enlargement of speckles was not as dramatic as the one seen for some cells in transient transfection experiments. This could be in part because there is no overexpression of the endogenous protein, even in the presence of TSA. We quantified the total SRSF2 signal in single cells and detected no significant difference between control and TSA-treated cells (Fig. 3B). By plotting the intensity profiles across a single nucleus, enrichment of endogenous SRSF2 in speckles can be seen as an increase in the height of the intensity peaks (Fig. S2). Even more clearly than its accumulation in speckles, depletion of splicing factors from the nucleoplasm in TSA-treated cells was evident from our experiments with endogenous SRSF2 (Fig. 3A). To quantify this effect in the cell population, we measured for each cell the intensity of the nucleoplasmic fraction and compared the distribution of the nucleoplasmic/total SRSF2 ratio (referred to as “nucleoplasmic fraction”) in control and TSA-treated cells. As shown in Figure 3C, cells treated with TSA showed a decrease in the SRSF2 nucleoplasmic fraction.

To study the structural organization of speckles and the nuclear distribution of SRSF2 at high resolution, we used Structured Illumination Microscopy (SIM)/OMX technique. TSA-treated cells showed speckles that were larger, more dense and with better defined edges than those of control cells (Fig. 3D). In addition, thanks to the SIM/OMX technology, we found that after TSA treatment speckles show a particular ordered internal structure similar to a repetitive tubular-like organization, as compared with control cells where speckles appear less ordered and structured (Fig. 3D, see high magnification insets; 3D-reconstruction of speckles in control and TSA-treated HeLa cells is shown in Movies S1 and S2, included as supplementary data). Furthermore, while speckles normally exhibit projections into the nucleoplasm that are considered as an evidence of recruitment of splicing factors to active transcription sites [19], the speckles of TSA-treated cells showed little continuity with the nucleoplasm. Indeed, the dispersed SRSF2 foci seen in the nucleoplasm and around speckles in control cells are diminished in TSA-treated cells (Fig. 3D, insets). In conclusion, TSA treatment causes depletion of endogenous SRSF2 from the nucleoplasm and results in its concentration in dense and well-structured speckles, with little continuity between these two compartments.

TSA treatment causes a massive increase in histone H3 and H4 acetylation (Fig. 2B) and general chromatin relaxation [45]. To test if the altered distribution of splicing factors can be induced by other types of chromatin structure perturbation and is not specifically related to histone acetylation, we analyzed the effect of knocking down the heterochromatin protein HP1α. This protein, like its paralog HP1γ, is necessary for chromatin-mediated increase in the inclusion of some alternative exons [32], [46]. This suggests that the chromatin conformation dependent on these proteins either participates in, or has cross-talk with, the splicing process. Efficient siRNA knockdown of HP1α in HeLa cells (Fig. S3 shows HP1α depletion) caused a redistribution of endogenous SRSF2 similar to that observed with the TSA treatment: depletion from nucleoplasm and accumulation in speckles (Fig. 3E). Again, this resulted in higher SRSF2 peaks when intensity profiles across a line are plotted (Fig. S4A) and in significantly increased SRSF2 signal associated to speckles when analyzing the cells population (Fig. S4B).

Together, our results indicate that a perturbation of the native chromatin structure leads to a redistribution of several splicing factors from the nucleoplasmic pool to speckles. These enlarged speckles are mostly discontinuous with the nucleoplasm, suggesting that recruitment of splicing factors from speckles to nascent pre-mRNA sites might be impaired.

### Dynamic Interactions are not Affected by Disruption of Chromatin Structure

**Figure 4:**
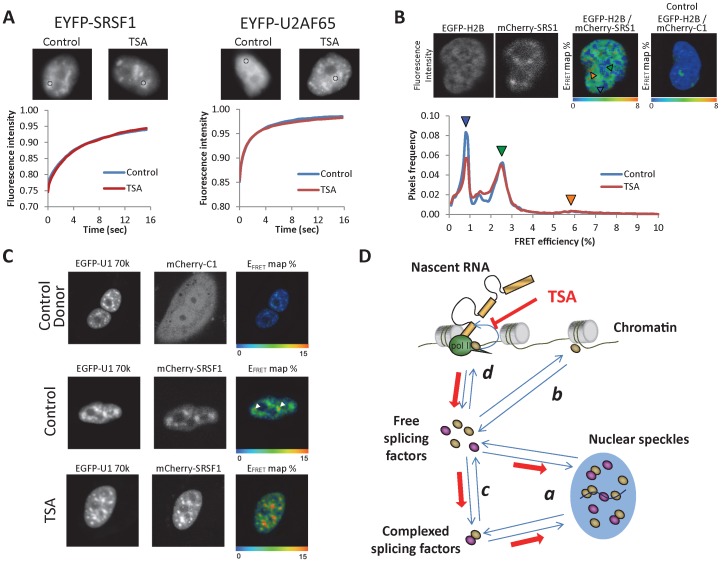
Splicing factor dynamics suggests that accumulation in speckles is due to inefficient recruitment to pre-mRNA. (A) FRAP analysis of SRSF1 and U2AF65 splicing factors fused to EYFP show that their kinetics of association to speckles are not modified after TSA treatment. For this experiment, C33A cells stably expressing the corresponding EYFP-fusion protein were used. Similar results were obtained for other splicing factors transiently transfected in HeLa cells (not shown). We show a representative cell for each group. The circle shows the speckle area where the laser pulse was applied, and where fluorescence recovery was measured afterwards. Fluorescence intensity is expressed relative to the fluorescence prior photobleaching. The length of TSA treatment was 4 h for U2AF65 and 6 h for SRSF1. The curves are averages from 10 to 15 cells. (B) Direct interaction between splicing factors and histones is detectable but not affected by TSA treatment. Interaction between the mCherry-tagged splicing factor SRSF1 and the EGFP-tagged histone H2B was analyzed by FLIM-FRET technique. A representative cell expressing both tagged proteins and its FRET efficiency map is showed. Also, the FRET efficiency map of a representative cell expressing EGFP-H2B and mCherry-C1 empty plasmid is shown as a control (NO FRET). The pixels histogram shows average FRET efficiency distributions for Control (7 cells) and TSA-treated cells (6 cells). Peaks of FRET are marked by colored arrowheads, indicating interaction between these two proteins. Nuclear regions associated to these distinct FRET populations are also marked by colored arrowheads in the E _FRET_ map. (C) Nucleoplasmic interaction between splicing factors is not impaired by TSA treatment. The interaction between EGFP-U1-70K and mCherry-SRSF1, a known interaction pair [51] was assessed by FLIM-FRET in Control and TSA-treated cells. Upper panels: control cells, transfected with mCherry instead of mCherry-SRSF1, show no FRET signal in either speckles or nucleoplasm. Lower panels: FRET efficiency between the two splicing factors is intermediate in nucleoplasm (green) with stronger (red) areas in speckles (marked with white arrowheads in control cells). (D) Proposed model to explain how relaxation of chromatin structure affects splicing factor distribution. Nucleoplasmic free splicing factors are in dynamic equilibrium with splicing factors in speckles (*a*). Free splicing factors can also interact with other splicing factors independently of splicing (*b*), these complexes being found both in nucleoplasm and speckles. However, what we call nucleoplasmic fraction involves also splicing factors briefly interacting with other molecules (such as histones) through abundant low affinity interactions (*c*) and splicing factors recruited to RNA and engaged in productive spliceosome assembly (*d*). For model simplicity, in *c* we only included interactions with histones and in *d* we only depicted co-transcriptional splicing. The red lines and arrows symbolize the proposed system response after TSA treatment: decreased efficiency in recruitment cause an excess of free splicing factors that is buffered by the speckles compartment.

To get further insight into the mechanism of splicing factor accumulation in speckles, we performed a series of microscopy experiments to measure changes in different aspects of splicing factor dynamics. For most of the experiments we used the SR protein SRSF1 as a model, because of the prior knowledge about its behavior and the availability of requisite tools. First, we measured the dynamics of SRSF1 association and diffusion from speckles by Fluorescence Recovery After Photobleaching (FRAP) experiments, either in untreated, or in TSA-treated cells (Fig. 4A, left). Little or no change in the rate of fluorescence recovery in speckles was observed after TSA treatment, suggesting that the affinities of the interactions responsible for the localization of this splicing factor in speckles are not affected. The same was observed for U2AF65 (Fig. 4A, right). This experiment provides strong evidence against the possibility that TSA exerts its effect through changing the interaction properties between splicing factors and their partners in speckles.

Splicing factors show a slower diffusion rate in the nucleoplasm than expected for freely diffusing molecules [47], [48], suggesting that they are continuously interacting with many components of the nucleus, including chromatin (either with DNA or histones), RNAs and other proteins. Therefore, a likely possibility is that the splicing factors interactions in the nucleoplasm are weaker after TSA treatment, increasing the number of free nucleoplasmic splicing factor molecules and therefore favoring their displacement to the speckles. Since TSA affects chromatin structure, we measure direct interaction between splicing factors and chromatin *in vivo*. Not much is known about how splicing factors interact directly with histones, besides one report of a phosphorylation-dependent interaction of SRSF1 and SRSF3 with histone H3 [49]. Bearing in mind that these interactions would possibly be weak (but nonetheless important for splicing factor distribution because of the abundance of histones), we used a sensitive method based on Förster Resonance Energy Transfer (FRET) by using Fluorescence Lifetime Imaging Microscopy (FLIM) technique [50] to detect and spatially map the direct interaction between EGFP-H2B histones and mCherry-SRSF1 splicing factors. In comparison to control E_FRET_ cell map (Fig. 4B, EGFP-H2B/mCherry-C1 panel), we were able to detect a widespread, although weak, direct interaction between these two proteins, which was not affected by TSA treatment (Fig. 4B, green signal in the EGFP-H2B/mCherry-SRS1 E_FRET_ cell map, marked by green arrow in the pixels histogram; see also Materials and Methods). An additional minor FRET efficiency population around 6% is detected between EGFP-H2B and mCherry-SRSF1 (see Fig. 4B, orange signal in the FRET cell map, marked by orange arrow in the pixels histogram) that might reflect EGFP-H2B/mCherry-SRSF1 interactions in a more constraint and tight chromatin environment. The interacting molecules are distributed through the nucleus but mostly excluded from speckles (Fig. 4B). This suggests that disruption of low affinity direct interactions between splicing factors and chromatin is not the cause of accumulation in speckles after TSA treatment.

As a model of the interaction among different splicing factors we used the known interacting partners U1 70K and SRSF1. These two splicing factors interact with each other in the nucleoplasm and in speckles, and some of the interacting pairs remain even in the absence of transcription and splicing [51]. We asked whether this direct interaction between different splicing factors in the nucleoplasm is diminished as a consequence of the TSA treatment. FLIM-FRET analysis of this interaction, either in the absence or presence of TSA, clearly argues against this hypothesis, since no decrease in FRET efficiency was detected in the nucleoplasm (Fig. 4C). On the contrary, the only effect observed was an increase of the red-colored areas in speckles (high-affinity interactions). Since these high-affinity interactions were already present in untreated cells (Fig. 4C, middle panels, marked by white arrowheads), but became more frequent after TSA treatment, this provided evidence of more interacting pairs formed upon chromatin perturbation. This is consistent with the accumulation in speckles seen in the previous experiments.

### Model of how the Perturbation of Chromatin Structure Affects Splicing Factor Distribution

The analysis of splicing factor behavior is complicated because of the many interactions, complexes and structures where they seem to be involved. Even a simplified model, as proposed in Figure 4D, has to take into account several equilibria. First, while a clear partition exists between the nucleoplasmic and the speckle-associated splicing factor pools, FRAP analysis shows that these two compartments are in dynamic equilibrium (marked as *a* in Fig. 4D). However, the nucleoplasmic fraction is not homogenous. Judging from analysis of diffusion by Fluorescence Correlation Spectroscopy (FCS) and FRAP experiments [47], [48], nucleoplasmic splicing factors do not diffuse freely, but rather are constantly engaged in interactions that reduce their effective diffusion rate. We exemplify these interactions with low affinity direct binding of splicing factor to histones (*b*), although it can also involve interactions with other proteins, with DNA and/or low affinity and non-productive binding to RNA. In addition, previous reports have shown the existence of complexes between different splicing factors that are at least partially splicing-independent [51]. These complexes exist both in the nucleoplasm and in speckles. We incorporate this in our model by adding an equilibrium between unbound nucleoplasmic splicing factors and nucleoplasmic splicing factors forming complexes (*c*), and also by assuming that complexed splicing factors are in equilibrium between nucleoplasm and speckles (second pair of arrows in *a*). We do not consider here the additional possibility of equilibrium between free and complexed splicing factors in speckles, but this would not affect our conclusions. Finally, in addition to free and complexed splicing factors, the nucleoplasmic pool also includes the splicing-engaged proteins and the ones that are forming stable complexes with mature mRNA. For simplicity, we only consider co-transcriptional recruitment, assuming also that the transcription machinery participates in the recruitment of splicing factors to nascent RNA (*d*).

**Figure 5:**
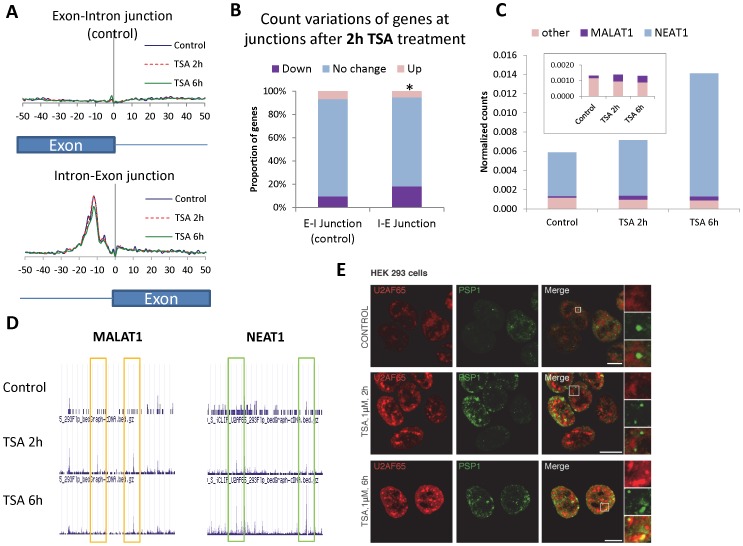
Chromatin relaxation affects spliceosome recruitment to nascent RNA and binding of splicing factors to ncRNAs. Binding of U2AF65 to RNA was assessed by iCLIP in 293-Flp cells, as a measure of spliceosome recruitment in Control cells or cells treated with 1 µM TSA for 2 h (before splicing factor accumulation in speckles is apparent) and 6 h. (A) TSA treatment causes an impairment of U2AF65 recruitment to the 3′ splice sites. Total U2AF65 counts in exon-intron (E-I) and intron-exon (I-E) junctions for all transcripts are plotted in a window of +/−50 nt from the junctions. The graph shows that iCLIP consistently captures U2AF65 at the expected location on the 3′ splice site (I-E junction), where only non-specific binding is detected at the E-I junction. The curve for 6 h TSA treatment (green) shows a reduction with respect to the control (blue) and 2 h TSA treatment (red). (B) A fraction of the genes show sensitivity to TSA treatment at 2 hours. Each junction was analyzed separately, and for each junction genes included in the analysis (see Materials and Methods) were divided into categories of increased (Up), decreased (Down) or unchanged (less than 2-fold change in any direction) U2AF65 counts in a region of +/−100 nt around the corresponding junction. E-I junction was used as a control, assuming that variations in these counts (non-specific) were random. Analysis of changes in the junctions of individual genes after 2 h TSA treatment shows that almost 20% of the genes tend to have less U2AF65 binding in I-E junctions (and only 5% have increased binding). As a control, E-I junction shows less than 10% of the genes with decreased binding and 7% with increased binding. Distributions for the two junctions are significantly different (asterisk, chi-squared test for independence between fold-change categories and type of junction, *p* = 0.00032). (C) U2AF65 binding to lincRNAs increase upon TSA treatment. Distribution of lincRNA iCLIP counts in the three libraries, discriminating by the abundant MALAT1 and NEAT1 nuclear-retained ncRNAs and all the other lincRNAs. Inset: magnification of the binding to MALAT1 and other lincRNAs, excluding NEAT1. (D) U2AF65 increase binding to specific sites in MALAT1 and NEAT1. UCSC browser window showing a portion of MALAT1 (top) and NEAT1 (bottom) genes and the U2AF65 iCLIP counts for Control, 2 h and 6 h TSA-treated cells (complete genes with binding sequence info can be found in Fig. S5). Orange boxes mark regions with peaks appearing at 2 h TSA and not further increasing, or even decreasing, at 6 h (MALAT1 peaks). Green boxes mark regions with peaks gradually increasing to their maximum at 6 h TSA (NEAT1 peaks). (E) Co-immunofluorescence analysis on untreated HEK-293 cells (top row) and cells treated with 1 µM TSA for 2 h (middle) or 6 h (bottom). For each condition, a high magnification view of speckle and paraspeckle compartments is shown (right panels). In untreated cells, the paraspeckles marker PSP1 does not colocalize with U2AF65 speckles, but after 6 h TSA co-localization of PSP1 and U2AF65 is observed. 2 h TSA treatment leads to an intermediate state were paraspeckles and U2AF65 granules are starting to be associated. Nuclei were stained with DAPI. Scale bars, 10 µm.

Based on our observations, we propose that the main cause for the observed changes in splicing factor distribution upon TSA treatment and HP1α knockdown is that productive recruitment of splicing factors to nascent RNAs is impaired by disruption of normal chromatin structure. We cannot distinguish here between two likely causes of reduced recruitment efficiency upon chromatin perturbation: either (a) less pol II pausing in the proximity of splice sites, or (b) modification of histone marks involved in the recruitment of splicing factors. Nevertheless, both explanations imply a participation of the chromatin structure in the splicing process. Several facts support this notion: 1) HP1α knockdown has similar effects to TSA treatment, suggesting that the effect is due to a change in chromatin structure (Fig. 3E) and not specifically to histone acetylation; 2) the distribution of not just one but several splicing factors is affected, suggesting a general impairment of the splicing process (Fig. 2A); 3) the splicing factors themselves are not acetylated in response to TSA treatment, implying that TSA is not affecting the proteins but rather a process in which the proteins take part, such as splicing (Fig. 2B); 4) while projections of speckles into the nucleoplasm are considered an indication of recruitment to transcription sites, the high resolution imaging analysis of TSA-treated cells shows speckle structures with little continuity with the nucleoplasm (Fig. 3D); 5) no changes in the kinetics of interchange between speckles and nucleoplasm could be detected by FRAP, suggesting that splicing factors are not being more strongly bound to a component of speckles (Fig. 4A); 6) while a decrease in nucleoplasmic interactions normally engaged by splicing factors could explain an excess of free protein (that is in turn relocated to speckles), no decrease was found after TSA treatment for the tested interactions (Fig. 4B and C).

Since splicing factors engaged in productive splicing are thought to form stable complexes with the mRNA, in most cases even being exported from the nucleus [52], [53], splicing commitment represents a drain of splicing factor from the equilibria depicted in Fig. 4D. Therefore, if a fraction of the splicing factors cease to be recruited (symbolized by the red inhibition symbol in Fig. 4D), the excess of free splicing factors will be buffered by the other compartments. Therefore, it is perfectly consistent that the excess of splicing factors not recruited for splicing is buffered by the speckles (this flow of the excess of free splicing factors is symbolized by the wider arrows in Fig. 4D). Since RNA bound splicing factors are detected in the nucleoplasmic pool, this model would explain the observed decrease in nucleoplasmic staining (Fig. 3C). In this case, we consider that the contribution of the splicing factor binding sites in chromatin and RNA molecules is minimal, which would be the case if these interactions have lower affinities than the ones that keep splicing factors in speckles. This assumption comes from the fact that the speckle compartment is known to buffer the excess of splicing factor when inhibiting transcription or splicing [18], [19], [20].

### TSA Impairs U2AF65 Recruitment to the 3′ Splice Sites of pre-mRNAs

To test the hypothesis that splicing factor accumulation in speckles is caused by a reduction in their commitment to splicing, we decided to measure whether recruitment of a splicing factor to pre-mRNA is affected by TSA treatment. We chose the U2-snRNP auxiliary factor protein U2AF65 for technical reasons, but also because it is a good indicator of early spliceosome assembly. As an unbiased approach, instead of measuring recruitment to a particular pre-mRNA, we used individual nucleotide resolution crosslinking immunoprecipitation (iCLIP) technique followed by high-throughput sequencing [54] to measure the recruitment of U2AF65 to bulk cellular pre-mRNAs. In this way we could monitor global spliceosome assembly efficiency, which can be affected by changes in the recruitment of many splicing factors, integrating the changes of multiple proteins in one output.

Based on the time course of splicing accumulation after TSA treatment (Fig. 2D), we performed two different time points for the iCLIP experiment: 2 hours, when the accumulation of splicing factor in speckles is starting, and 6 hours, when the accumulation of splicing factors in speckles is at its maximum. As shown in Figure 5A, while no accumulation U2AF65 is detected in any region of exon-intron junctions (E-I, top), a peak near the end of introns. i.e. at intron-exon junctions (I-E, bottom) is present in the three different conditions, consistent with the fact that U2AF65 binds to the poly-pyrimidine tract upstream of 3′splice sites (3′ss). This also provides evidence for the specificity of the iCLIP experiment. A comparison of the types of RNA sequences represented in the three libraries showed that there are no major differences between the Control and the TSA samples in the distribution of U2AF65 binding among types of RNA sequence (Fig. S5A). When we analyzed counts on specific RNA biotypes, the only category where a clear increase is seen upon TSA treatment is the long intergenic non-coding RNAs (Fig. S5B, “lincRNA”, marked with an orange box).

When comparing the heights of the 3′ss peaks among the three samples (Control, TSA 2 h and TSA 6 h) a decrease of around 17% in the height of the 3′ splice site peak is detected for TSA 6 h, but not for TSA 2 h (Fig. 5A bottom, green line), showing that recruitment of U2AF65 to 3′ss is diminished after 6 h of TSA treatment. This result supports our model, since 6 hours after TSA addition splicing factor depletion from the nucleoplasm is evident from the microscopy experiments.

Since our model indicates that failure of splicing factor recruitment is the cause of the accumulation, a partial reduction in U2AF65 binding to 3′ splice sites is expected at the 2 h point. This could not be evident in the bulk of pre-mRNA bound U2AF65 (Fig. 5A), but we assume that some genes and exons would be more sensitive to the TSA effect than others. We therefore analyzed the proportion of genes where U2AF65 binding to intron-exon junctions was affected by the 2-hour TSA treatment. Our iCLIP experiment has not enough sequencing depth to perform a complete analysis on a single exon basis, but we were able to extract a number of genes where the count number in the I-E junctions was significant enough to analyze changes between the libraries (see legend of Figure 5B and Materials and Methods).

We analyzed the proportion of genes where the normalized I-E count number was either increased or decreased by at least 2-fold changed in any direction after TSA treatment. As a control for changes in the non-specific binding of U2AF65 we used a similar criterion to analyze E-I junctions. Figure 5B shows that while nearly 20% of the genes show decreased U2AF65 binding at the I-E junction in TSA 2 h, only 5% display increased binding, while most of the genes show no change in any direction above the 2-fold threshold. As a control, genes that change in the E-I junction are not biased towards any direction, as expected from non-specific binding that is not associated to the splicing process. As indicated in the figure, the difference between the distributions is statistically significant. This result indicates that even at an early time (2 hours) the effect of TSA decreasing the efficiency of splicing factor recruitment in a subset of genes can be detected, although this decrease is still not visible when analyzing the bulk of U2AF65 binding to 3′ss.

Since 3′ splice sites differ considerably between genes in mammals, the fact that a fraction of the genes are more susceptible to alterations in the chromatin structure is expected. Also, genome-wide studies of nucleosome distribution showed that exons flanked by longer introns and weak splice sites have a stronger nucleosome positioning [39], [42]. Based on this, we could speculate that weak 3′ss (for example those with shorter poly-pyrimidine tracts) may need participation of chromatin assisted recruitment mechanisms to enhance their recognition efficiency. However, as previously mentioned, the sequencing depth of our experiment precludes us to statistically analyze exon-by-exon to undercover patterns of exon sensitivity to TSA.

### The Speckle-localized Non-coding RNA MALAT1 Binds U2AF65 Rapidly After TSA Treatment

As mentioned previously, RNA molecules participate in nuclear compartmentalization, either with structural or regulatory roles. Nuclear retained ncRNAs such as MALAT1 or NEAT1 bind splicing factors and can regulate their localization and function. In the case of speckles, MALAT1 is located in these granules and modulates the localization of several splicing factors [25], [26] and their phosphorylation status [25]. MALAT1 seems to be particularly abundant in neuronal cells, where it plays a role in gene expression [26]. Also, the splicing factor TDP43 seems to be sequestered by the ncRNAs MALAT1 and NEAT1 in the brain cells of individuals with frontotemporal lobar degeneration, where the level of these transcripts is augmented [55].

Since the only RNA sequence biotype that appeared over-represented after TSA treatment in our iCLIP experiment was long intergenic non-coding RNAs (lincRNAs), we asked whether this could reflect binding of splicing factors to MALAT1. First, we analyzed which lincRNAs showed increased binding after TSA treatment. As seen in Fig. 5C, U2AF65 bound to MALAT1 and NEAT1 explain almost completely the increase seen in total binding to lincRNAs after 2 h and 6 h-TSA time points, with NEAT1 having between 80–90% of all lincRNA counts. However, the kinetics of U2AF65 binding to these ncRNAs is different: while binding to MALAT1 increases at 2 h and then stays roughly at the same level at 6 h of TSA (see inset), binding to NEAT1 only increases modestly at 2 h but shows a pronounced increase in binding at 6 h of TSA.

We next looked at the sites where U2AF65 binding to these ncRNAs increases. Consistent with the variations in total counts, new peaks (representing U2AF65 binding sites) appear in the case of MALAT1 upon 2 h TSA treatment and either remain at the same height, or are lost at the 6 h time point (Fig. 5D and Fig. S6, top, orange boxes). The sequences correspond to short poly-pyrimidine stretches, which likely are low-affinity U2AF65 binding sites. In the case of NEAT1, the U2AF65 binding peaks increase only partially at 2 h TSA and are significantly higher at 6 h (Fig. 5D and Fig. S5, bottom, green boxes), consistent with what we observed for total lincRNA counts (Fig. 5C).

Since MALAT1 binding of U2AF65 is correlated with concentration of splicing factors in speckles, this re-localization could be explained by either an increase in speckle localization or in total levels of this ncRNA. First, we analyzed the localization of MALAT1 ncRNA in untreated or TSA treated cells using fluorescence in situ hybridization (RNA-FISH). After 2 hours of TSA treatment, when the increase in binding of U2AF65 is detected by iCLIP, MALAT1 speckle-distribution remained comparable to that of control cells (Fig. S7), arguing against a causative role of MALAT1 in splicing factor localization. At 6 h time point, MALAT1 showed a decreased localization in speckles (Fig. S7). This MALAT1 reorganization also observed after transcription inhibition [26], suggesting that it is secondary to the accumulation of splicing factors in speckles, since Figures 2C and S1 clearly show that TSA causes no inhibition of general transcription. In addition, we analyzed the steady state levels of this ncRNA in HeLa cells treated with TSA for 6 hours, using quantitative RT-PCR. We observed that MALAT1 levels were not affected by the treatment (Fig. S8, left). These experiments strongly suggest that changes in MALAT1 are not the cause of splicing factor re-localization, supporting the notion that this ncRNA mainly acts as a buffer of free splicing factors in the nucleus.

In contrast to MALAT1, NEAT1 steady state levels increase around 50% (Fig. S8, right) after 6 h TSA treatment. Therefore, augmented levels of NEAT1 after TSA treatment could explain the observed increase of NEAT1-bound U2AF65, which is also seen at the 6-hour time point. Since this ncRNA is a structural component of paraspeckles [24], we investigated the possibility of a stronger association of U2AF65 with these particular nuclear bodies. Interestingly, co-immunostaining of endogenous U2AF65 and the paraspeckle protein PSP1 [56] in HEK-293 cells show that, while U2AF65 was not co-localized with paraspeckles in control cells, after 6 hours of TSA treatment the paraspeckles greatly increased their association to U2AF65 speckles (Fig. 5E, see also magnifications at the right). At 2 hour time point, the association was partial and weaker (Fig. 5E). The same observation was done in HeLa cells (Fig. S9).

In summary, while these results support a direct relationship between splicing factor re-localization and binding to MALAT1, they strongly suggest that the accumulation of splicing factors in the granular compartment is not due to increased presence of MALAT1 in nuclear speckles. Since the total levels of MALAT1 remain similar after TSA, the appearance of new U2AF65 binding events on low affinity sites should be related to the re-localization of U2AF65 from the nucleoplasm to speckles and can be interpreted as a buffering interaction that deals with the excess of free U2AF65. Whether the presence of such sites accounts for the capacity of speckles to accommodate the excess of splicing factors remains to be explored for this and other splicing factors. In the case of NEAT1, an increase in expression levels could account for more U2AF65 binding after 6 h TSA treatment. This suggests an alternative mechanism to the model presented in Fig. 4D to explain the re-distribution of splicing factors, in this case from nucleoplasm to paraspeckles. However, this association with paraspeckles involved a minor fraction of U2AF65 (Fig. 5E and Fig. S9), suggesting that most of the splicing factor concentration in speckles is not related to the increase in expression of NEAT1 and the association with paraspeckles. Of course, it doesn’t exclude the possibility that both mechanisms are acting together and that, for example, the decrease of U2AF65 binding to the bulk of 3′ splice sites at 6 h (Fig. 5A) could be mediated in part by sequestration of U2AF65 by NEAT1.

### Conclusions

It has been known for some time that HDAC inhibitors can affect alternative splicing choices, since the first report of TSA counteracting the effect of plasmid replication on alternative splicing of a fibronectin exon reporter [34]. In the light of further mechanistic studies that demonstrated that slower pol II elongation favors recognition of weak exons [57], this result was interpreted as an effect of chromatin structure on pol II processivity: relaxation of chromatin prevents pol II to pause, giving less time for efficient co-transcriptional recognition of weak splice sites. Further evidence was presented for this model in endogenous genes [32], [33], [35].

We should remark that although changes in chromatin structure were shown to affect alternative splicing by promoting separate changes in pol II elongation and splicing factor recruitment, both mechanisms can be considered together in a single model for co-transcriptional regulation of alternative splicing. In fact, it is completely plausible that the changes in pol II elongation determine the efficiency of splicing factor recruitment to specific RNA elements, in particular if recruitment is mediated by the transcriptional machinery. An important part of this hypothesis is that recruitment of splicing factors is, at least for some events, inefficient and can be helped by pol II pausing. In a previous study with the HDAC inhibitor sodium butyrate, where splicing microarray analysis was used to uncover ∼700 genes with alternative splicing events sensitive to histone acetylation, it was shown that the treatment can affect the recruitment of the SR protein SRSF5 (previously known as SRp40) to one of the responsive exons [58]. However, the example was limited to a single protein in a single pre-mRNA species, which prevented generalization of the mechanism.

Here, we present evidence that chromatin relaxation affects the distribution of multiple splicing factors. By using a range of imaging approaches, we demonstrate that this effect likely reflects a decrease in the association of splicing factors with RNA, which can affect spliceosome recruitment in a subset of exons. Why some exons are particularly sensitive to chromatin perturbation remains to be resolved, although this is consistent with results showing that only a small fraction of the alternatively spliced genes assayed for sensitivity to sodium butyrate was in fact affected by the treatment [58]. It seems that some splicing events have a stronger need for chromatin assistance than others. The mechanistic reasons for this await further molecular analysis of the dynamic interactions between splicing factors and specific RNA substrates.

## Materials and Methods

### Plasmids

Plasmids expressing fluorescent fusion proteins were based on EGFP-C1, EYFP-C1 (Clontech Laboratories, Inc) or mCherry-C1 [59] expression vectors. Plasmids expressing mCherry-H2B [45], EGFP-U1 70K, mCherry-SRSF1, EYFP-SRSF3 [51], EGFP-U1A [60], T7-SRSF1 and T7-SRSF2 proteins were previously described [61]. EGFP-SRSF1 is a generous gift from G. Biamonti (Istituto di Genetica Molecolare (IGM-CNR). EGFP-SRSF2 construct was generated by subcloning SRSF2 fragment from mCherry-SRSF2 as described previously [48]. EGFP-U2AF65 construct was generated by EcoR1/BamH1 digestion and gel extraction from EYFP-U2AF65 [62] and ligation into pEGFP-C1. pSV2-mMALAT1 [25] construct was a kind gift from Dr. KV. Prasanth, University of Illinois at Urbana-Champaign, USA.

### Cell Culture, Treatments and Transfections

HeLa, HEK-293T and N2a cells were cultured in DMEM (Invitrogen) supplemented with 10% FBS and 100 U/ml penicillin/streptomycin. The stable HeLa cell line expressing histone H2B-EGFP [45] was cultured in DME supplemented with 10% FBS, 100 U/ml penicillin/streptomycin, and 2 µg/ml blasticidin-S. TSA (Sigma-Aldrich) was added to the cells at a final concentration of 0.2 ng/µl for 6 hours, or otherwise indicated. Depolarization treatment of N2a cells consisted in supplementing the culture medium with 60 mM KCl for 6 hours. Cells were treated with 5,6-Dichloro-1-beta-D-ribofuranosylbenzimidazole (DRB) at 25µg/ml for 2 h. For actinomycin D (ActD) treatment, cells were incubated with 1 µg/ml ActD for 2 h.

C33A cells were grown in 6 well dishes and transfected with EYFP-U2AF65 and EYFP-SRSF1 constructs following Effectene transfection reagent instructions (Qiagen). 24 hours after transfection, cells were put under selection with G418 at 200 µg/ml in DMEM supplemented with 10% FCS. After 3–4 weeks of selection, resistant colonies were picked and screened for expression of the fluorescently tagged proteins. Clones showing acceptable levels of expression were picked and seeded into 24 well dishes and grown under selection for a further 2 weeks. The clones were screened by fluorescence microscopy and colonies showing expression of the tagged proteins in all cells were expanded as cell lines.

When indicated, cells were transfected with 1 µg/90-mm dish of the appropriate plasmid DNA using Effectene (QIAGEN) transfection reagent (HeLa) or Lipofectamine 2000 (Invitrogen) reagent (N2a and HEK-293T) according to the manufacturer’s instructions. For siRNA transfections in HeLa cells, the RNAiMAX reagent (Invitrogen) was used. siRNA against HP1α, a kind gift from Dr. Alexia Ferrand (Wellcome Trust Biocentre, Dundee), was previously described [63].

C33A and N2a cells were originally obtained from ATCC.

### Imaging of Fixed and Living Cells

For imaging on fixed cells, cells were grown on glass coverslips and fixed for 5 min in 3.7% paraformaldehyde in 37°C PHEM buffer (60 mM Pipes, 25 mM Hepes, 10 mM EGTA, and 2 mM MgCl2, pH 6.9). After a 10-min permeabilization with 1% Triton X-100 in PBS, cells were blocked with 1% goat serum for 30 min and then incubated with primary antibodies for 1 h, washed, and incubated with secondary antibodies for 45 min. If required, cells were stained with 0.3 µg/ml DAPI (Sigma-Aldrich). After a final set of washes, cells were mounted in Vectashield media (Vector Laboratories). Primary antibodies: anti-SRSF1 (S4045, Sigma) antibody was used 1∶1000, anti–HP1α (Euromedex) was used 1∶500, anti-U2AF65 (mAb MC3, Sigma) was used 1∶100 and anti-PSP1 [56] was used 1∶1000. anti-H3K36me3 (abcam 9050) was used at 1∶500.

For live imaging of FP-fused proteins, cells were cultured in glass-bottomed dishes (WILCO; Intracel). Before imaging, growth medium was replaced with phenol red–free CO2-independent medium (Invitrogen).

Immunofluorescence images were acquired with a wide-field fluorescence microscope (DeltaVision Spectris; Applied Precision) and a CoolMax charge-coupled device camera (Roper Industries). Imaging was performed at room temperature using a 60× oil immersion NA 1.4 Plan-Apochromat objective from Olympus. SoftWoRx software (Applied Precision) was used for image acquisition. We used a deconvolution cluster (Spinlock) for offline deconvolution of data. For co-localization and FISH experiments, fluorescence images were acquired using a laser scanning confocal microscope LSM780 from Zeiss (63×1.4 NA oil Plan-Apochromat objective, Zeiss). Z-series of 0.4 µm slices spanning the entire nuclei were recorded. ImageJ software was used for image analysis.

### 5-FU Incorporation Assay

HeLa cells, either mock treated or ATP depleted for 30 min or after washing and recovery for 30 min, were incubated with 2 mM 5-FU (Sigma-Aldrich) for 30 min at 37°C. Subsequently, cells were fixed, permeabilized, and incubated with primary anti-BrdU antibody (1∶500; Sigma-Aldrich). Immunofluorescence microscopy was performed as indicated in the previous section. Mean intensity values were used for quantification and analysis.

### Quantification of Speckles Intensity

We measured the splicing factor presence in speckles for EGFP-SFSR1 and 2 (Fig. 1C) and endogenous SFSR2 (Fig. S3B). Quantification of the fluorescence signal in single cells was performed after acquisition, using ImageJ software. The focal plane used for quantification was selected by comparing the mean, standard deviation and maximum values of the stack and selecting the highest. Speckles were selected using Maximum Entropy threshold, and then the integrated density of all speckles was calculated using particle analysis. For correcting for cell-to-cell variations in size or signal intensity, speckles values were normalized to the total integrated density of that cell.

### Quantification of Total Endogenous SRSF2 and SRSF2 Nucleoplasmic Fraction

Quantification of the fluorescence signal in single cells was performed after acquisition, using ImageJ software. Since several focal (z) planes were acquired for each field, the first step when analyzing a single cell was to measure the mean intensity of anti-SRSF2 fluorescence in all z planes to find the maximum value, to be sure that in each cell the image used has a well-focused nucleus. In that z plane, we used ImageJ Default threshold option to select the nucleus keeping the areas with background fluorescence out of the calculation. This threshold value was named *A*. The integrated density value applying this threshold corresponded to total SRSF2 signal. By measuring DAPI signal in the same z plane, we can quantify total SRSF2 signal relative to DAPI signal. Then, we used ImageJ Maximum Entropy threshold option, which resulted in selection of the high signal intensity areas of the images, in this case, the speckles. This threshold value was named *B*. Finally, using *A* and *B* values to manually adjust down and up thresholds, we calculated the intermediate SRSF2 signal, which corresponded to nucleoplasmic signal. The nucleoplasmic fraction was calculated by dividing nucleoplasmic/total SRSF2 signal ((*A*-*B*)/*B*).

### Structured Illumination Protocol for Image Acquisitions

The protocol was as described previously [64]. 3DSIM was performed on a microscope system OMX version 2 (Applied Precision, Issaquah, WA) equipped with 405, 488, and 593 and 642 nm solid-state lasers. Images were acquired using a UPlanS Apochromat 100×1.4 NA oil immersion objective lens and back-illuminated 512×512 electron microscopy charge-coupled device cameras (Cascade II; Photometrics). Samples were illuminated by a coherent scrambled laser light source that had passed through a diffraction grating, thus generating interference of light orders in the image plane to create a 3D sinusoidal pattern with lateral stripes ∼0.2 µm apart. The pattern was shifted laterally through five phases and three angular rotations of 60° for each z section. Optical sections were separated by 0.125 µm. Exposure times were between 200 and 500 ms, and the laser power was adjusted to achieve optimal intensities between 2000 and 4000 counts in a raw image of 16-bit dynamic range at the lowest laser power possible to minimize photobleaching. Each frame acquisition was separated by a 300-ms pause. Multichannel imaging was achieved through sequential acquisition of wavelengths by separate cameras. Raw 3DSIM images were processed and reconstructed to reveal structures with greater resolution [65]. The channels were aligned in x, y and rotationally by using predetermined shifts as measured using a target lens and the SoftwoRx alignment tool (Applied Precision). After correction for image shifts due to differences in emission wavelengths, data sets were transferred to SoftWoRx software (Applied Precision) for visualization and generation of xz plane images.

### Fluorescence Recovery After Photobleaching (FRAP)

C33A stable cell lines or HeLa cells were directly cultured or transiently transfected in glass-bottomed dishes (WILCO; Intracel), respectively. Before imaging, growth medium was replaced with phenol-red free CO2- independent medium (Invitrogen). Cells expressing FP-fusion proteins were imaged using Olympus U-Plan-Apo 100×1.35NA objective and photobleached by using the “photokinetic experiment” function of the DeltaVision Spectris microscope. Briefly, a small region inside nucleus was photobleached with a 488 nm laser (100% laser power for the duration of 0.15 s), and time-lapse sequences of single optical sections for imaging FP-fluorescence were collected with an exposure time of 0.05 s for each image. The fluorescence intensities in the bleached and non-bleached area before (three time points) and after laser photobleaching were quantified using ImageJ software.

### Fluorescence Lifetime Imaging Microscopy

FLIM–FRET measurements were acquired and analyzed as described previously [50], [51]. Briefly, FRET measurements by FLIM were performed on a confocal laser-scanning microscope (Radiance 2100 MP; Bio-Rad Laboratories) on a stand (TE2000; Nikon) using a 60×/1.4 NA Plan Apo oil immersion lens (Nikon) equipped with a titanium sapphire laser (Chameleon from Coherent) providing femtosecond pulses at a 90-MHz repetition rate. Light shielding and environmental control were achieved using a black environmental chamber that surrounded the microscope stage and stand (Solent Scientific) maintaining cells at 37°C and limiting stray light from entering the detectors. Presence of both EGFP and mCherry signals was confirmed using the confocal light path with the 488-nm argon ion and 543-nm HeNe laser lines. Two photon excitation for FLIM was performed at 890 nm with a 600 fps scan speed at 512×512 resolution for 90–120 s, and fluorescent light was collected on a nondescanned detector (5783P; Hamamatsu Photonics) using a 670-nm long-pass dichroic mirror and a 528/50-nm band-pass emission filter, such that only GFP was excited and collected. Time correlated single photon counting was performed using a photon-counting card (SPC830; Becker & Hickl), and subsequent analysis was performed with SPCImage (Becker & Hickl).

Because FRET interactions cause a decrease in the fluorescence lifetime of the donor molecules (EGFP), the FRET efficiency can be calculated by comparing the FLIM values obtained for the EGFP donor fluorophores in the presence and absence of the mCherry acceptor fluorophores (Fig. 4 B and Fig. 4C). Mean FRET efficiency images were calculated such as the FRET efficiency, E_FRET_ = 1 - (τDA/τD), where τ DA is the mean fluorescence lifetime of the donor (H2B-EGFP or EGFP-U1 70K) in the presence of the acceptor (mCherry-SRF1) and τD is the mean fluorescence lifetime of the donor (H2B-EGFP EGFP-U1 70K) expressed in the presence of mCherry-C1 acceptor for all of the cells imaged. In the non-FRET conditions, the mean fluorescence lifetime value of the donor in the absence of the acceptor was calculated from a mean of the τD by applying a monoexponential decay model to fit the fluorescence lifetime decays. Then, in the FRET conditions, we applied a biexponential fluorescence decay model to fit the experimental decay. We obtained information about the lifetime of two populations of molecules, i.e., the noninteracting donor population τD and the donor population that was interacting with the acceptor (τDA) as well as the intensity factors *a* and *b* of the two decay components. By fixing the noninteracting proteins lifetime τD using data from control experiments (in the absence of FRET), the value of τDA was estimated. Then, by knowing both values τDA and τD at each single pixel, the FRET efficiency (E_FRET_) was derived by applying the following equation: E_FRET_ = 1 - (τDA/τD) at each pixel in a selected ROI using SPCImage software. Furthermore, the FRET distribution curves (Fig. 4 B, bottom panel) from these ROIs were displayed from the extracted associated matrix using SPCImage software.

### RNA Fluorescence in Situ Hybridization (RNA-FISH)

To detect MALAT1 RNA, HeLa cells were grown on glass coverslips, rinsed briefly with PBS and fixed in 4% formaldehyde in PBS for 15 min at RT. Then, cells were permeabilized in PBS containing 0.5% Triton X-100 and 5 mM vanadyl-ribonucleoside complex (VRC, Sigma) on ice for 10 min; washed with PBS 3×10 min and rinsed once in 2×SSC prior to hybridization. Hybridization was carried out using nick-translated full-length mMALAT1 cDNA probe (Abbott Molecular, USA) in a moist chamber at 37°C overnight as described elsewhere [25]. Fluorescence images were acquired as described above.

### SR Protein Acetylation Assay

HEK-293T cells transfected with T7-SRSF1, T7-SRSF2 or empty vector were lysed in 293 IP Buffer (20 mM Tris PH = 7.5, 150 mM KCl, 1% Triton X-100, 1 mM EDTA, 1 mM EGTA). Lysates were clarified 10′ at 12.000 RPM. Immunoprecipitation was performed overnight with T7 tag antibody-agarose (Novagen), washed 3 times and resuspended in Laemmli Buffer. Western blot was done against anti-T7 tag monoclonal antibody (1∶10000, Novagen) or anti-acetylated lysine antibody (1∶1000, Cell Signalling). Histones were extracted from cells and western blot with anti-total H3 antibody (Upstate, Millipore) was performed as in [35]. Where mentioned, 10 µM MG132 (Sigma) was used.

### U2AF65 iCLIP Analysis

iCLIP experiment was performed as previously described [54]. We used a HEK-293T cell line harboring a FRT site (Flp-in 293, Invitrogen). We prepared libraries from three samples: non-treated cells (control), cells treated with 1 µM trichostatin A for 2 hours (TSA 2 h) or 6 hours (TSA 6 h). For immunoprecipitation, 7 µl of monoclonal anti-U2AF65 antibody (U4758, Sigma) was used for each sample. A no-antibody sample was also included. iCLIP data analysis was carried on through the iCount pipeline by the Blaz Zupan lab in the University of Ljubljana (http://icount.biolab.si).

For analysis of the proportion of genes with change of U2AF65 binding in exon-intron (E-I) or intron-exon (I-E) junctions after 2-hour TSA treatment, a list of genes was made for each junction (including +/−100 nt from the proper junction) containing the genes with count number above threshold for at least one library. Since TSA 2 h library was approximately 5-fold larger than Control library, the threshold was set in 10 counts for Control and 50 counts for TSA 2 h. 742 genes were included in the I-E junction group and 600 genes in the E-I junction group. The number of counts per gene in each library was normalized using the total number of counts in the same library. Then, we excluded genes from the two groups that showed more than 2-fold change in normalized total counts in the whole gene (not restricted to junctions), assuming that the change in total counts evidenced that mRNA levels of these genes were affected by the TSA treatment, making interpretation of the variations difficult. After filtering out these genes 565 genes were included in the I-E group and 461 in the E-I group. The E-I group, of comparable size than the I-E group, was used as a control for non-specific U2AF65 binding variations, since Fig. 5A shows that counts are not associated to any region of the junction but rather homogenously distributed. Genes of the two groups were categorized as Up (if normalized junction counts were at least 2-fold higher in TSA 2 h than in Control library), Down (same but lower in TSA 2 h than in Control library) and No Change (less than 2-fold change in the normalized junction counts between TSA 2 h and Control libraries). For statistical evaluation, we used chi-squared test for independence, considering the Up-Down-No Change categories as a variable and the type of junction (E-I or I-E) as the other. The null hypothesis was that the distribution of genes in the categories Up-Down-No Change was independent from the type of junction. To reject this hypothesis would imply that U2AF65 binding to the I-E junctions (splicing associated binding) responds differently to TSA than to the E-I junctions (non-specific binding).

### Measurement of lincRNAs Levels

Total RNA extraction from cells, retrotranscription and real time PCR was performed as previously described, including amplification of the HSPCB housekeeping gene mRNA [35], except that random decamers were used for RT instead of oligo-dT primer. Sequence primers for MALAT1 and NEAT1 amplification was obtained from [25].

## Supporting Information

Figure S1 **No alteration of H3K36me3 after TSA treatment.** Immunostaining experiments using antibody against histone H3 lysine 36 (H3K36me3), a hallmark of pol II transcriptional elongation, on HeLa cells untreated or treated with TSA 2 h and 6 h. A statistical quantification of anti-H3K36me3 fluorescence intensity is shown. No changes were observed after TSA treatment. Nuclei were counter-stained with DAPI. Scale bars, 10 µm.
(TIFF)

Figure S2 **Endogenous SRSF2 accumulates in speckles after TSA treatment.** Analysis of intensity profile of endogenous SRSF2 and H3 levels signals across a line (yellow dotted lines) for representative control and TSA-treated HeLa cells. SRSF2 profile shows lower basal intensity and higher peaks in TSA-treated cells, corresponding to depletion of SRSF2 from nucleoplasm and accumulation in speckles. No changes are seen for H3 profiles. The wide valley of H3 signal in the TSA-treated cell profile corresponds to the presence of a nucleolus.
(TIF)

Figure S3 **Assessment of HP1α knockdown efficiency in HeLa cells.** HP1α protein was detected by immunofluorescence with a specific antibody in cells transfected with either a control siRNA or a siRNA targeting HP1α mRNA. DAPI staining of the same field is shown to observe the cell nuclei. Scale bar, 10 µm.
(TIF)

Figure S4 **Knockdown of HP1α causes a similar effect on endogenous SRSF2 distribution as TSA treatment.** (A) SRSF2 localization after HP1α knockdown. HeLa cells were transfected with control or HP1α-specific siRNAs. After three days, they were fixed and immunostained for endogenous SRSF2 detection. DAPI staining is also shown. Cells transfected with HP1α siRNA show less nucleoplasmic staining and more intense speckles than control cells. For each sample, a representative cell and the analysis of intensity profile of endogenous SRSF2 across the indicated line are shown. (B) Statistical analysis of endogenous SRSF2 enrichment in speckles after HP1α depletion. Signal of SRSF2 in speckles increases in siHP1α transfected cells vs. cells transfected with control siRNA. Intensity of splicing factor in all speckles of a focal plane was calculated for individual cells using automatic threshold and particle analysis (see Materials and Methods). The total integrated density of speckles particles was normalized by total integrated density of the cell. *n* = 21 and 27 for siControl and siHP1 respectively. * means significant differences between treated and control cells, using Mann-Whitney U test (p = 0.0021) or Kolmogov-Smirnov test (p = 0.022).
(TIF)

Figure S5 **Comparison between the iCLIP libraries.** (A) Distribution of cDNA counts between the different types of sequences in the Control, TSA 2 h and TSA 6 h libraries. 3UTR: 3′ untranslated regions; 5UTR: 5′ untranslated regions; ORF: open reading frames; inter: intergenic regions; intron: intronic regions; ncRNA: non-coding RNAs; telo: telomeric sequences; as: counts mapped to anti-sense sequences. (B) Distribution of cDNA counts among sequences biotypes. The only clear tendency is the increase in “ncRNA, lincRNA” category (orange box) upon TSA treatment.
(TIF)

Figure S6 **U2AF65 binding sites on MALAT1 and NEAT1 ncRNAs.** UCSC browser window showing the complete MALAT1 (top) and NEAT1 (bottom) genes and the U2AF65 iCLIP counts for Control, 2 h and 6 h TSA-treated cells. As in Fig. 5D, orange boxes mark regions with peaks appearing at 2 h TSA and not further increasing, or even decreasing, at 6 h (MALAT1 peaks). Green boxes mark regions with peaks gradually increasing to their maximum at 6 h TSA (NEAT1 peaks). For all the boxed peaks, the corresponding sequence is shown, with an arrowhead pointing to the binding nucleotide.
(TIF)

Figure S7 **Changes in MALAT1 localization upon TSA treatment are not correlated with changes in splicing factor localization or binding.** RNA FISH using mMALAT1 probe in control untreated or 1 µM TSA-treated HeLa cells. MALAT1, as expected, shows a speckled pattern in control HeLa cells. A 2 h TSA treatment does not lead to any visible change in this pattern, while at this time U2AF65 binding to MALAT1 increases (Fig. 5C). Moreover, a 6 h treatment results in partial re-distribution of MALAT1 ncRNA from nuclear speckles to nucleoplasm, while at this time-point the splicing factors are concentrated in speckles (Fig. 2A and D; Fig. 3A). These observations suggest that MALAT1 is not causing the re-distribution of splicing factors in the nucleus. Note that weak levels of MALAT1 can still be observed in some nuclear speckles. DNA is counterstained with DAPI in blue. Right panels show higher magnifications of individual nuclei. Scale bar, 10 µm.
(TIF)

Figure S8 **NEAT1 but not MALAT1 RNA levels are affected by TSA treatment.** Total RNA was extracted from HeLa cells, either untreated or incubated with 1 µM TSA for 6 hours. Steady-state levels of MALAT1 and NEAT1 ncRNAs were analyzed by RT followed by quantitative real time PCR. The MALAT1 and NEAT1 levels are relativized to the mRNA levels of the housekeeping gene HSPCB. No differences were found between the two samples for MALAT1, while a significant increase is detected in TSA samples for NEAT1 (Student’s *t* test, *p* = 0.926 for MALAT1 and *p* = 0.0176 for NEAT1, marked by asterisk).
(TIF)

Figure S9 **Gradual increase in U2AF65 splicing factor and paraspeckles colocalization after TSA treatment in HeLa cells.** Same as Fig. 5E, but for HeLa cells. Co-immunofluorescence of HeLa control cells (top row) and cells treated 1 µM TSA for 2 h (middle) or 6 h (bottom). For each condition, a high magnification view of speckle and paraspeckle compartments is shown (right panels). In untreated cells, the paraspeckles marker does not colocalize with U2AF65 speckles but, after 6 h TSA, co-localization of PSP1 and U2AF65 is observed. 2 h TSA treatment leads to an intermediate state were paraspeckles and U2AF65 are starting to be associated. Nuclei were stained with DAPI. Scale bars, 10 µm.
(TIF)

Movie S1 **3D reconstruction of individual speckles in control cells using the SIM/OMX high-resolution microscopy system (see also** **Figure 3D** **in the main article).**
(MPG)

Movie S2 **3D reconstruction of individual speckles in TSA-treated cells using the SIM/OMX high-resolution microscopy system (see also** **Figure 3D** **in the main article).**
(MPG)
