# Supplementary figures and images for: Perturbation of Chromatin Structure Globally Affects Localization and Recruitment of Splicing Factors
Source: PLoS One. 2012 Nov 12;7(11):e48084. doi: 10.1371/journal.pone.0048084 (PMC3495951; doi:10.1371/journal.pone.0048084)

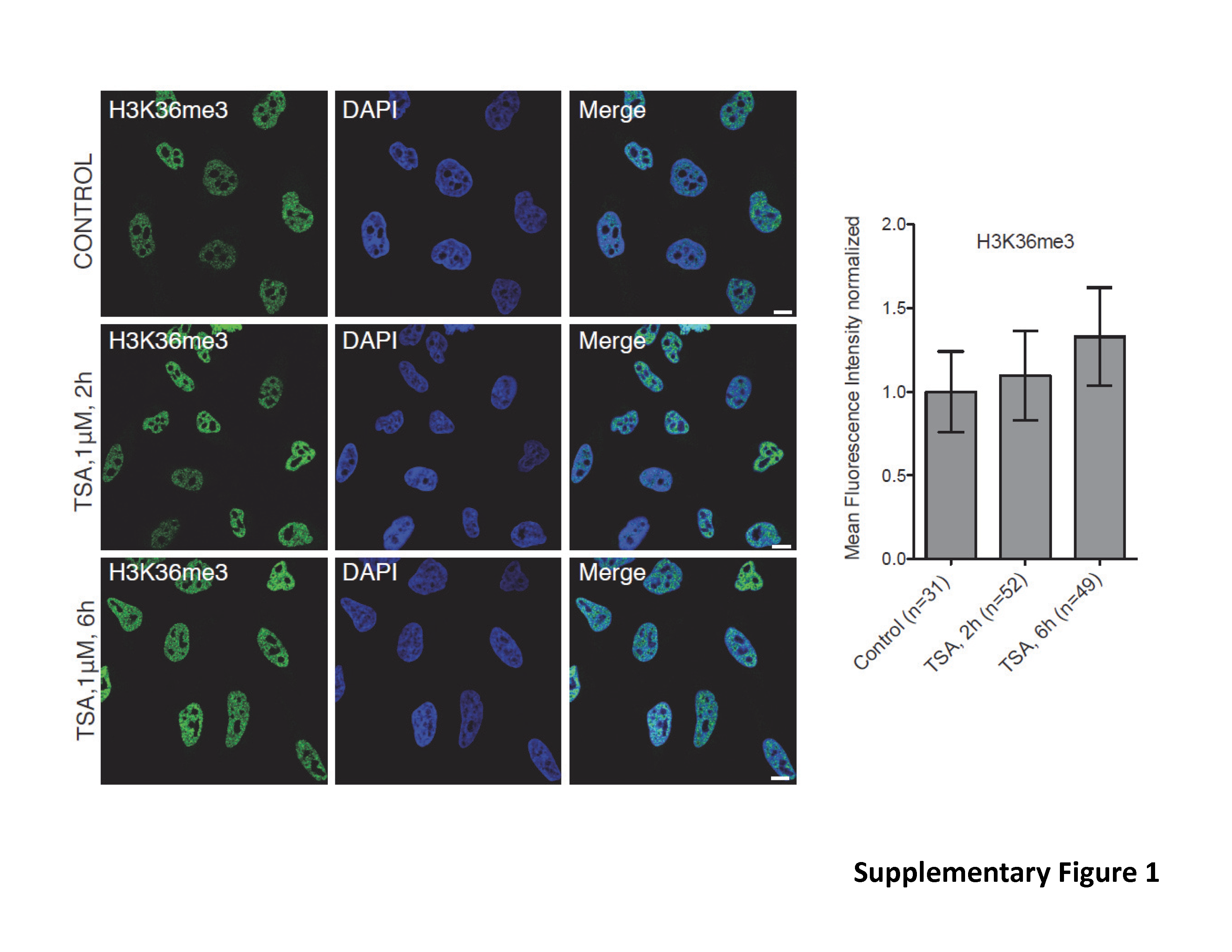

Supplement: Figure S1 — No alteration of H3K36me3 after TSA treatment. Immunostaining experiments using antibody against histone H3 lysine 36 (H3K36me3), a hallmark of pol II transcriptional elongation, on HeLa cells untreated or treated with TSA 2 h and 6 h. A statistical quantification of anti-H3K36me3 fluorescence intensity is shown. No changes were observed after TSA treatment. Nuclei were counter-stained with DAPI. Scale bars, 10 µm. (TIFF) [file pone.0048084.s001.tif]

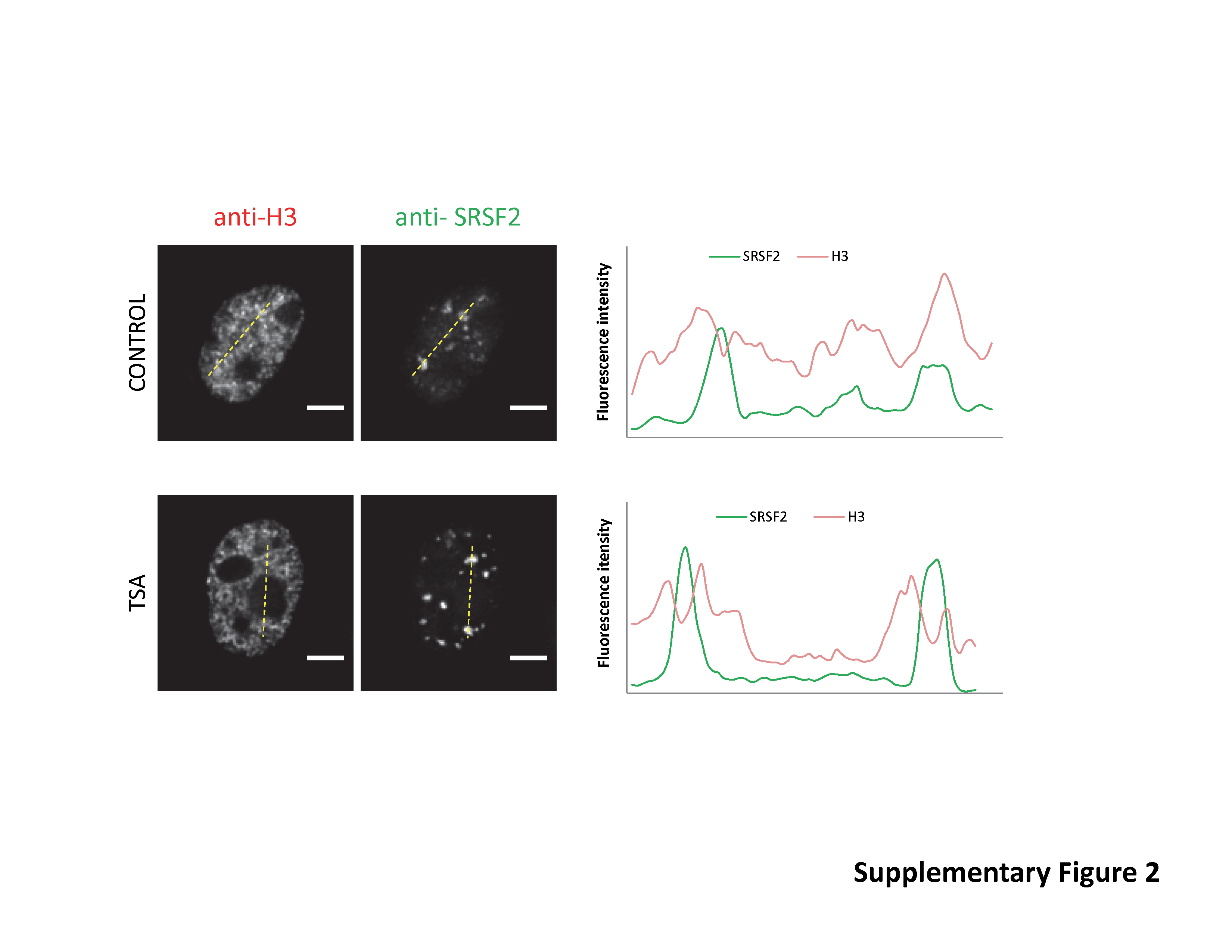

Supplement: Figure S2 — Endogenous SRSF2 accumulates in speckles after TSA treatment. Analysis of intensity profile of endogenous SRSF2 and H3 levels signals across a line (yellow dotted lines) for representative control and TSA-treated HeLa cells. SRSF2 profile shows lower basal intensity and higher peaks in TSA-treated cells, corresponding to depletion of SRSF2 from nucleoplasm and accumulation in speckles. No changes are seen for H3 profiles. The wide valley of H3 signal in the TSA-treated cell profile corresponds to the presence of a nucleolus. (TIF) [file pone.0048084.s002.tif]

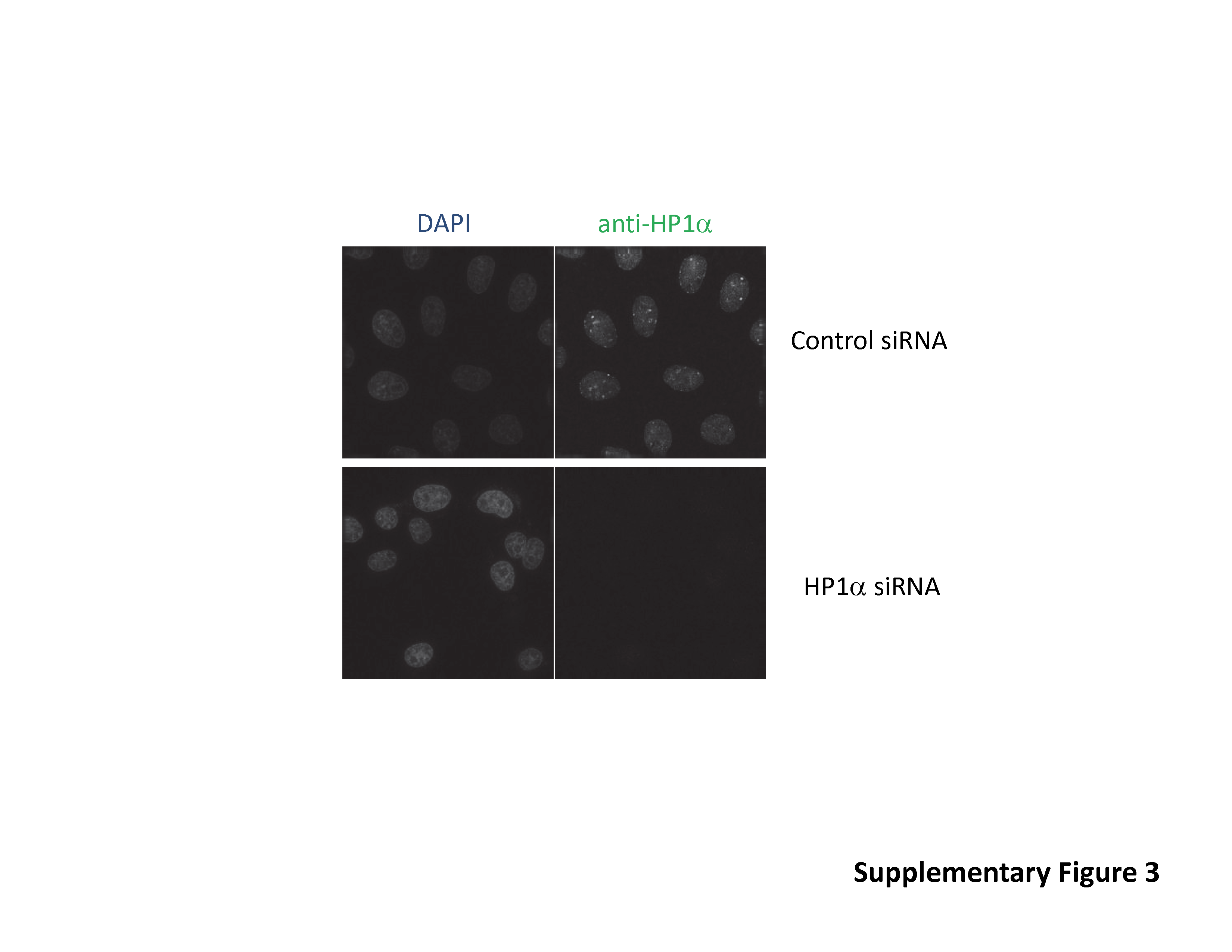

Supplement: Figure S3 — Assessment of HP1α knockdown efficiency in HeLa cells. HP1α protein was detected by immunofluorescence with a specific antibody in cells transfected with either a control siRNA or a siRNA targeting HP1α mRNA. DAPI staining of the same field is shown to observe the cell nuclei. Scale bar, 10 µm. (TIF) [file pone.0048084.s003.tif]

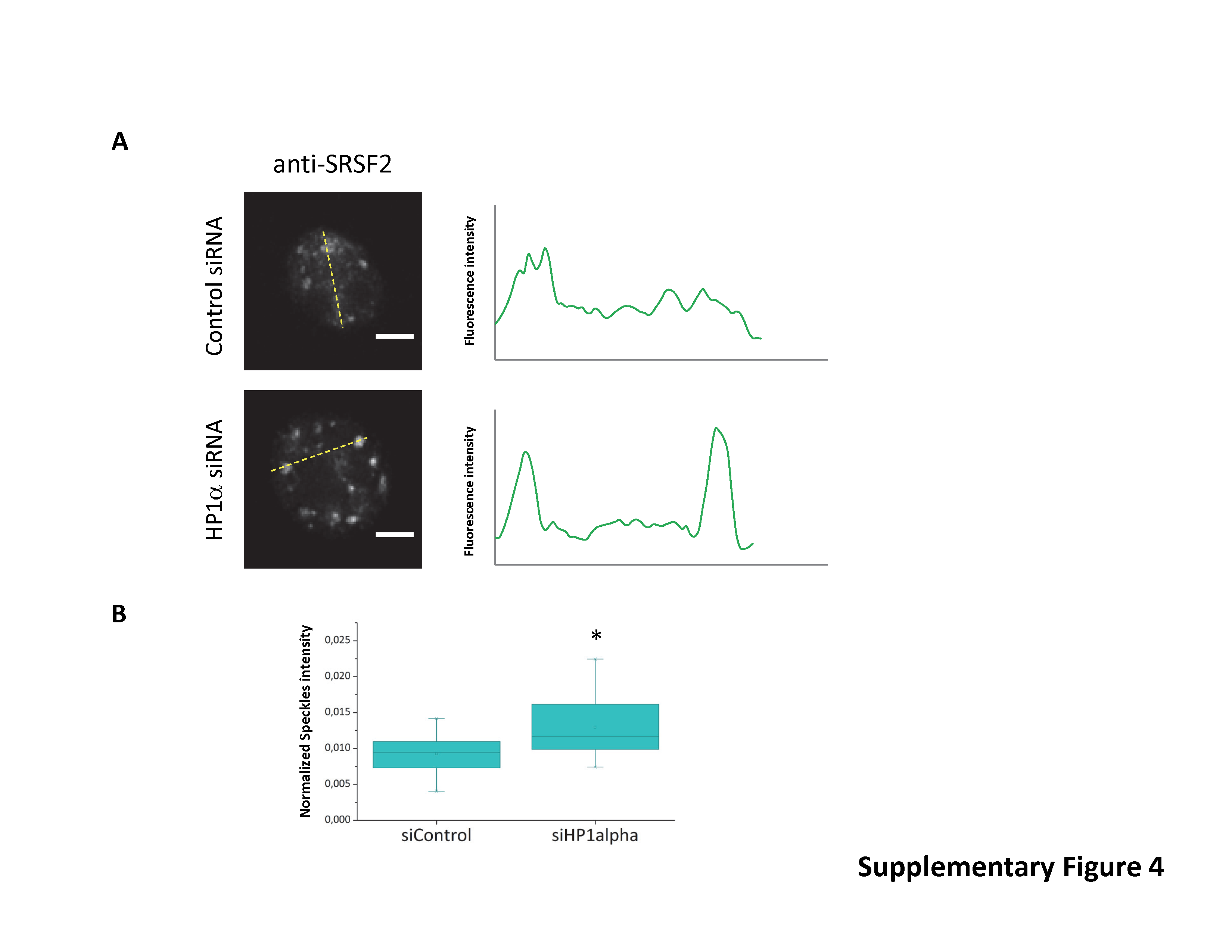

Supplement: Figure S4 — Knockdown of HP1α causes a similar effect on endogenous SRSF2 distribution as TSA treatment. (A) SRSF2 localization after HP1α knockdown. HeLa cells were transfected with control or HP1α-specific siRNAs. After three days, they were fixed and immunostained for endogenous SRSF2 detection. DAPI staining is also shown. Cells transfected with HP1α siRNA show less nucleoplasmic staining and more intense speckles than control cells. For each sample, a representative cell and the analysis of intensity profile of endogenous SRSF2 across the indicated line are shown. (B) Statistical analysis of endogenous SRSF2 enrichment in speckles after HP1α depletion. Signal of SRSF2 in speckles increases in siHP1α transfected cells vs. cells transfected with control siRNA. Intensity of splicing factor in all speckles of a focal plane was calculated for individual cells using automatic threshold and particle analysis (see Materials and Methods). The total integrated density of speckles particles was normalized by total integrated density of the cell. n = 21 and 27 for siControl and siHP1 respectively. * means significant differences between treated and control cells, using Mann-Whitney U test (p = 0.0021) or Kolmogov-Smirnov test (p = 0.022). (TIF) [file pone.0048084.s004.tif]

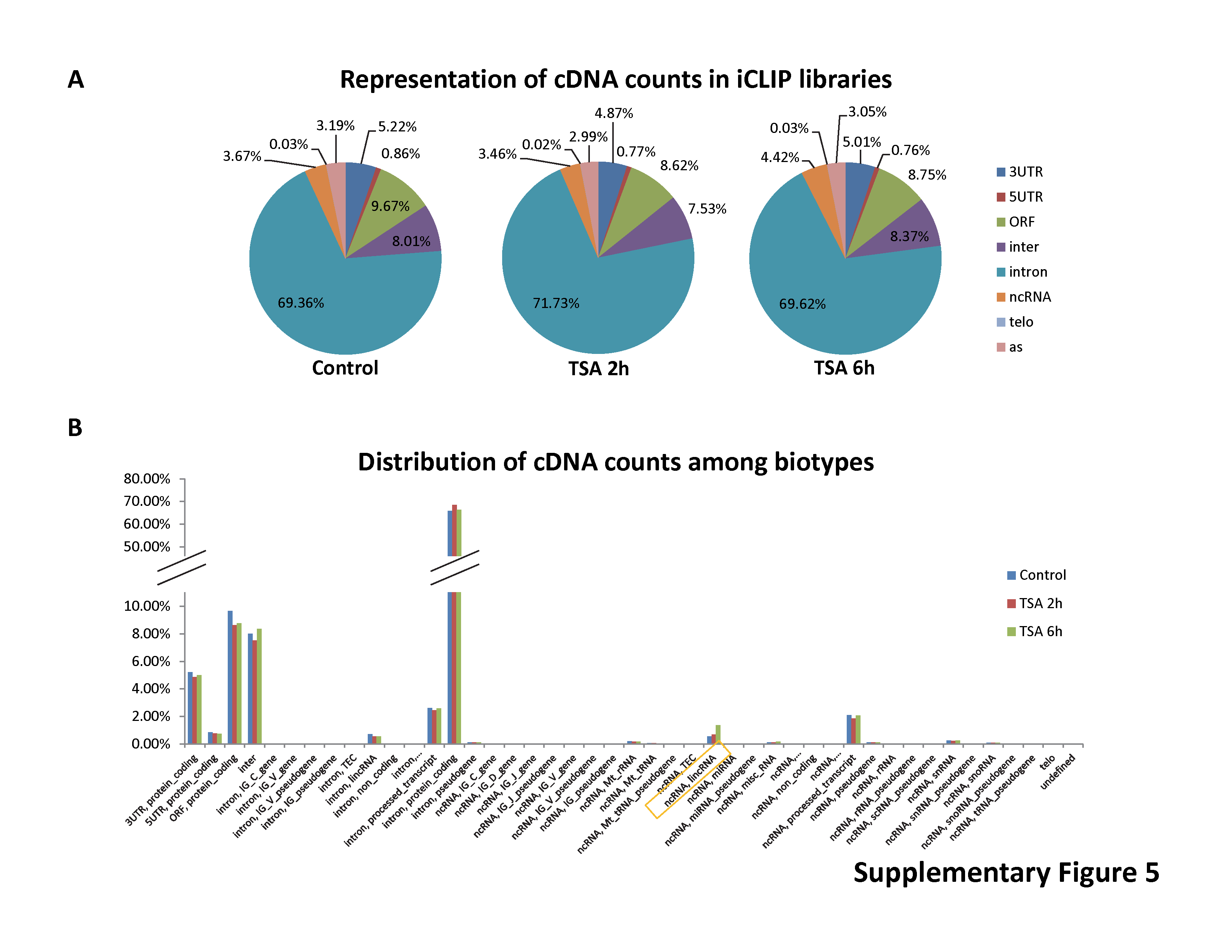

Supplement: Figure S5 — Comparison between the iCLIP libraries. (A) Distribution of cDNA counts between the different types of sequences in the Control, TSA 2 h and TSA 6 h libraries. 3UTR: 3′ untranslated regions; 5UTR: 5′ untranslated regions; ORF: open reading frames; inter: intergenic regions; intron: intronic regions; ncRNA: non-coding RNAs; telo: telomeric sequences; as: counts mapped to anti-sense sequences. (B) Distribution of cDNA counts among sequences biotypes. The only clear tendency is the increase in “ncRNA, lincRNA” category (orange box) upon TSA treatment. (TIF) [file pone.0048084.s005.tif]

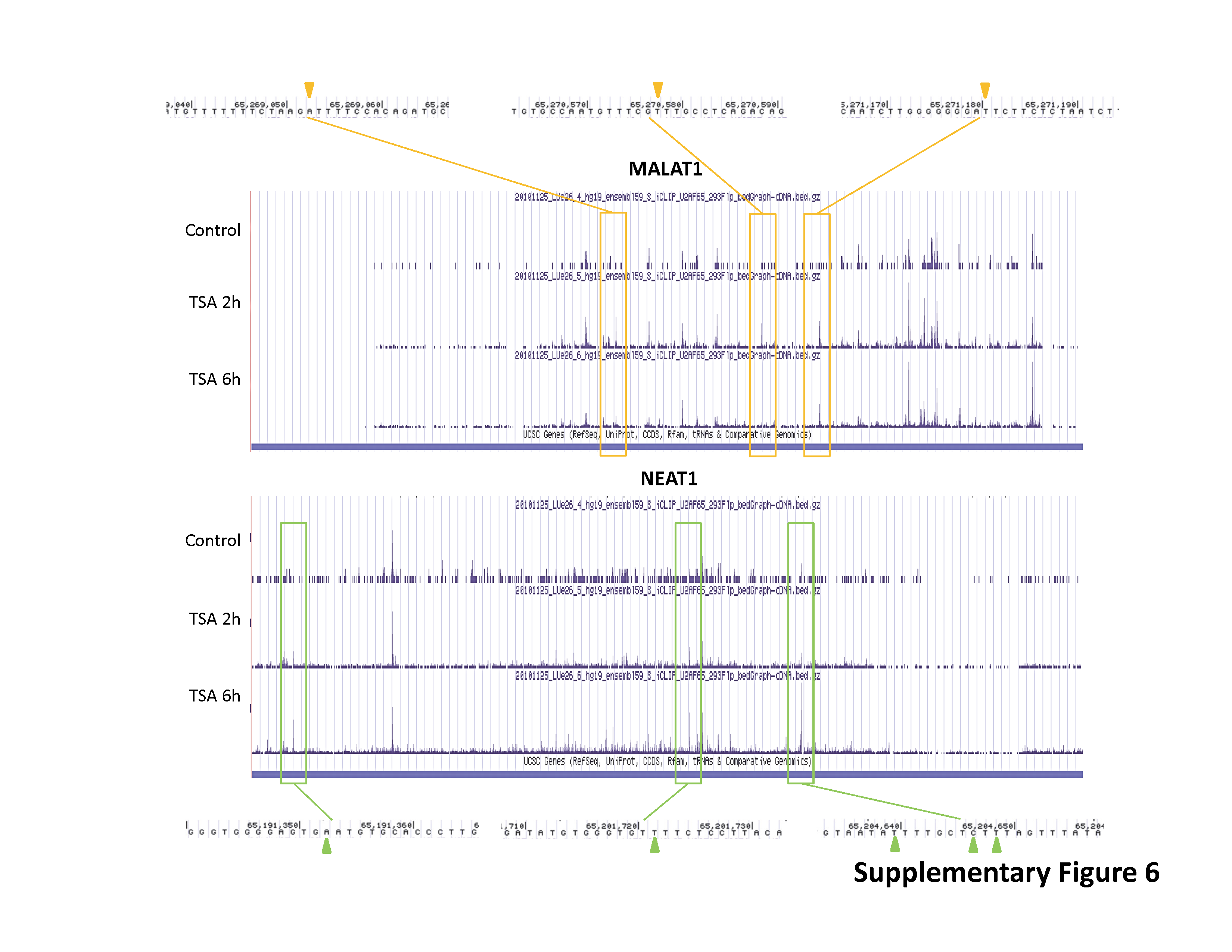

Supplement: Figure S6 — U2AF65 binding sites on MALAT1 and NEAT1 ncRNAs. UCSC browser window showing the complete MALAT1 (top) and NEAT1 (bottom) genes and the U2AF65 iCLIP counts for Control, 2 h and 6 h TSA-treated cells. As in Fig. 5D, orange boxes mark regions with peaks appearing at 2 h TSA and not further increasing, or even decreasing, at 6 h (MALAT1 peaks). Green boxes mark regions with peaks gradually increasing to their maximum at 6 h TSA (NEAT1 peaks). For all the boxed peaks, the corresponding sequence is shown, with an arrowhead pointing to the binding nucleotide. (TIF) [file pone.0048084.s006.tif]

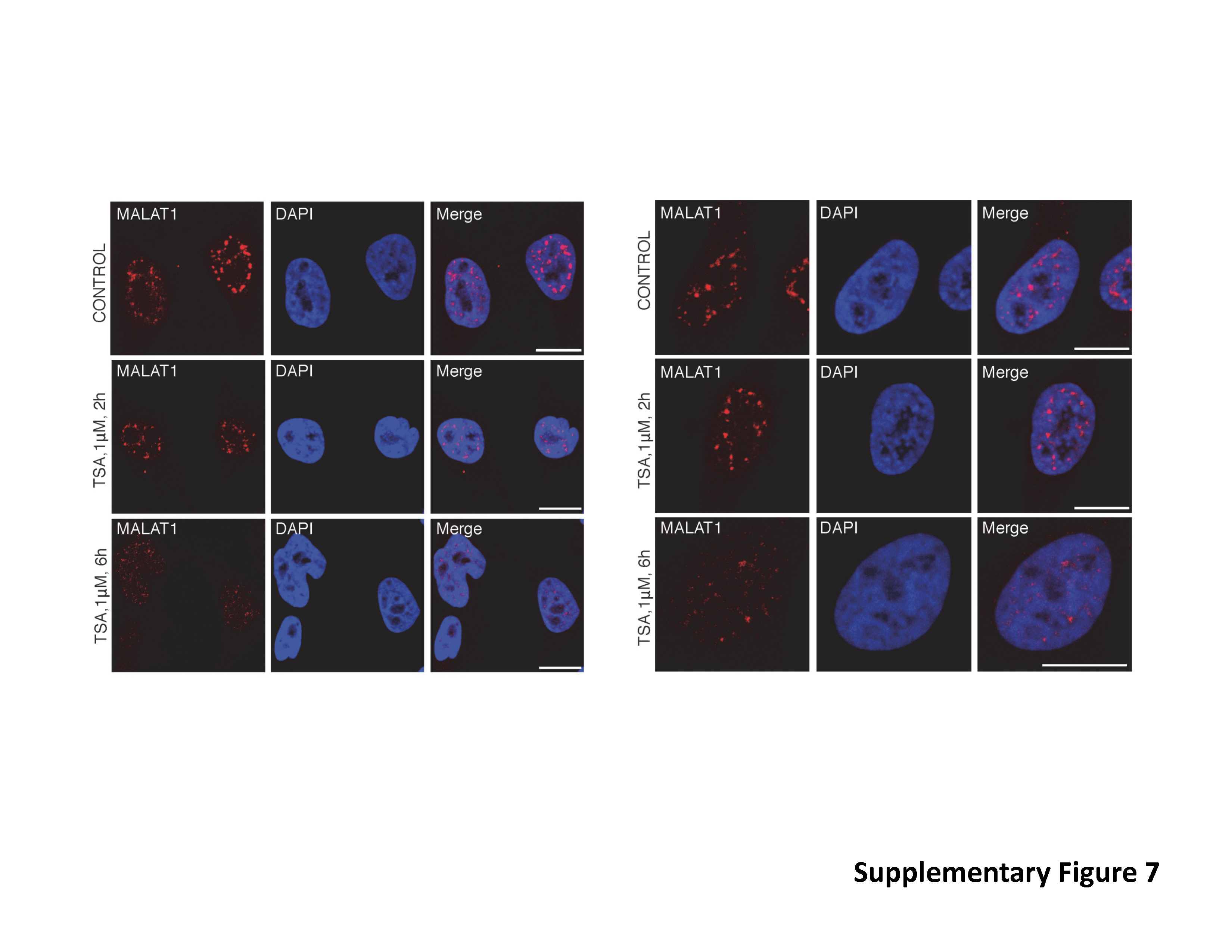

Supplement: Figure S7 — Changes in MALAT1 localization upon TSA treatment are not correlated with changes in splicing factor localization or binding. RNA FISH using mMALAT1 probe in control untreated or 1 µM TSA-treated HeLa cells. MALAT1, as expected, shows a speckled pattern in control HeLa cells. A 2 h TSA treatment does not lead to any visible change in this pattern, while at this time U2AF65 binding to MALAT1 increases (Fig. 5C). Moreover, a 6 h treatment results in partial re-distribution of MALAT1 ncRNA from nuclear speckles to nucleoplasm, while at this time-point the splicing factors are concentrated in speckles (Fig. 2A and D; Fig. 3A). These observations suggest that MALAT1 is not causing the re-distribution of splicing factors in the nucleus. Note that weak levels of MALAT1 can still be observed in some nuclear speckles. DNA is counterstained with DAPI in blue. Right panels show higher magnifications of individual nuclei. Scale bar, 10 µm. (TIF) [file pone.0048084.s007.tif]

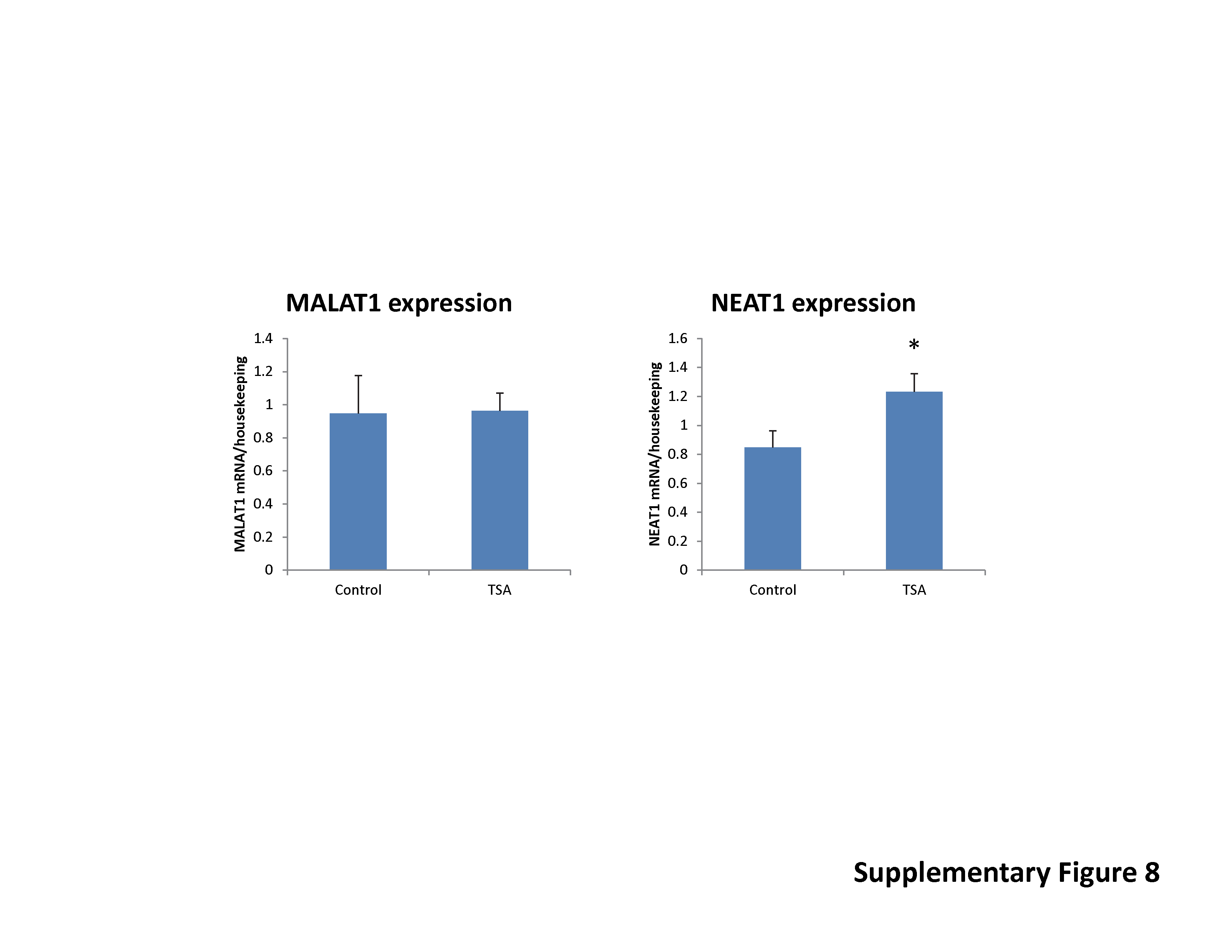

Supplement: Figure S8 — NEAT1 but not MALAT1 RNA levels are affected by TSA treatment. Total RNA was extracted from HeLa cells, either untreated or incubated with 1 µM TSA for 6 hours. Steady-state levels of MALAT1 and NEAT1 ncRNAs were analyzed by RT followed by quantitative real time PCR. The MALAT1 and NEAT1 levels are relativized to the mRNA levels of the housekeeping gene HSPCB. No differences were found between the two samples for MALAT1, while a significant increase is detected in TSA samples for NEAT1 (Student’s t test, p = 0.926 for MALAT1 and p = 0.0176 for NEAT1, marked by asterisk). (TIF) [file pone.0048084.s008.tif]

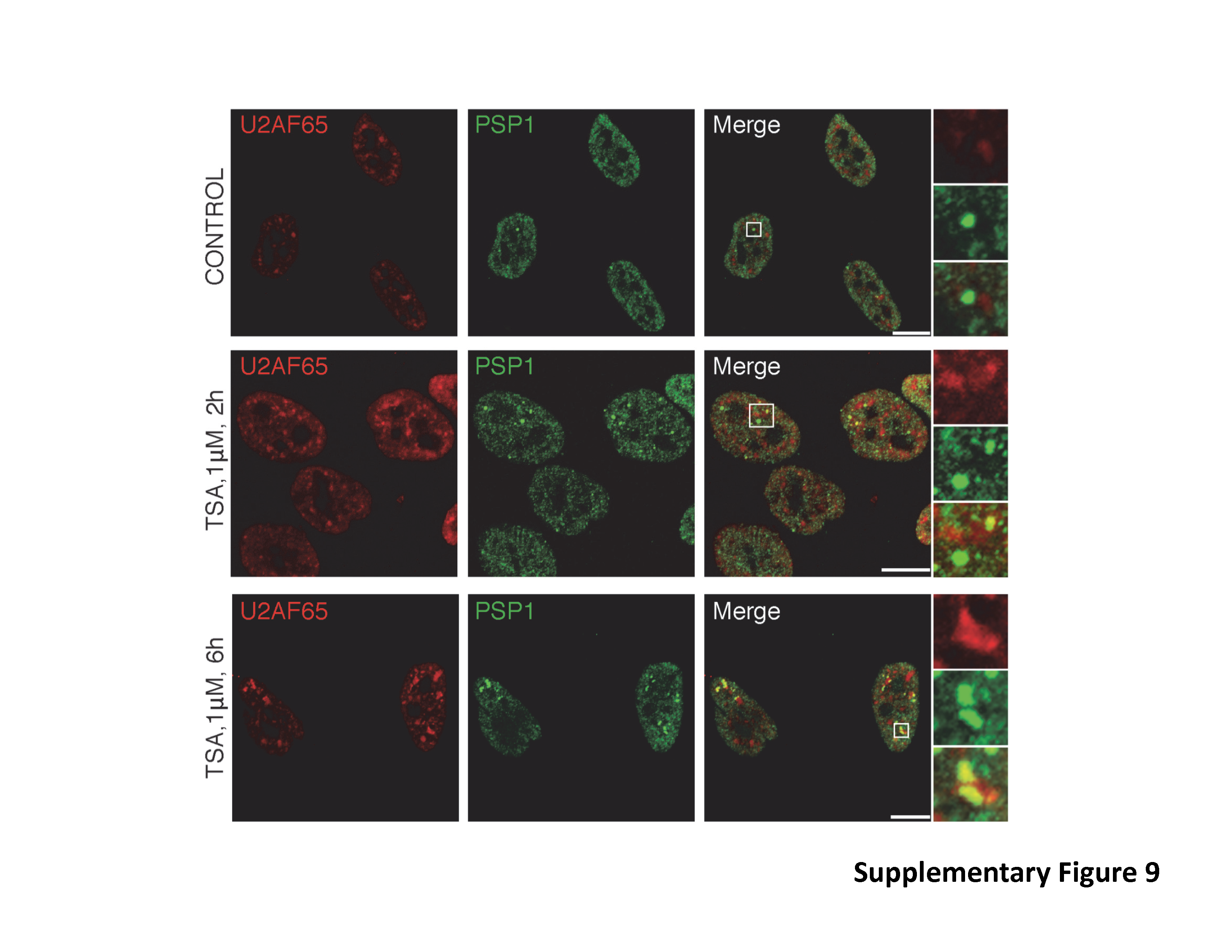

Supplement: Figure S9 — Gradual increase in U2AF65 splicing factor and paraspeckles colocalization after TSA treatment in HeLa cells. Same as Fig. 5E, but for HeLa cells. Co-immunofluorescence of HeLa control cells (top row) and cells treated 1 µM TSA for 2 h (middle) or 6 h (bottom). For each condition, a high magnification view of speckle and paraspeckle compartments is shown (right panels). In untreated cells, the paraspeckles marker does not colocalize with U2AF65 speckles but, after 6 h TSA, co-localization of PSP1 and U2AF65 is observed. 2 h TSA treatment leads to an intermediate state were paraspeckles and U2AF65 are starting to be associated. Nuclei were stained with DAPI. Scale bars, 10 µm. (TIF) [file pone.0048084.s009.tif]
